# Exome sequencing of lymphomas from three dog breeds reveals somatic mutation patterns reflecting genetic background

**DOI:** 10.1101/gr.194449.115

**Published:** 2015-11

**Authors:** Ingegerd Elvers, Jason Turner-Maier, Ross Swofford, Michele Koltookian, Jeremy Johnson, Chip Stewart, Cheng-Zhong Zhang, Steven E. Schumacher, Rameen Beroukhim, Mara Rosenberg, Rachael Thomas, Evan Mauceli, Gad Getz, Federica Di Palma, Jaime F. Modiano, Matthew Breen, Kerstin Lindblad-Toh, Jessica Alföldi

**Affiliations:** 1 Broad Institute, Cambridge, Massachusetts 02142, USA;; 2 Science for Life Laboratory, Department of Medical Biochemistry and Microbiology, Uppsala University, Uppsala SE 751 23, Sweden;; 3 Dana-Farber Cancer Institute, Boston, Massachusetts 02215, USA;; 4 North Carolina State University, Raleigh, North Carolina 27695, USA;; 5 Harvard Medical School, Boston, Massachusetts 02115, USA;; 6 Massachusetts General Hospital, Boston, Massachusetts 02114, USA;; 7 Animal Cancer Care and Research Program, College of Veterinary Medicine, and Masonic Cancer Center, University of Minnesota, Minneapolis, Minnesota 55455, USA;; 8 University of North Carolina Lineberger Comprehensive Cancer Center, Chapel Hill, North Carolina 27514, USA

## Abstract

Lymphoma is the most common hematological malignancy in developed countries. Outcome is strongly determined by molecular subtype, reflecting a need for new and improved treatment options. Dogs spontaneously develop lymphoma, and the predisposition of certain breeds indicates genetic risk factors. Using the dog breed structure, we selected three lymphoma predisposed breeds developing primarily T-cell (boxer), primarily B-cell (cocker spaniel), and with equal distribution of B- and T-cell lymphoma (golden retriever), respectively. We investigated the somatic mutations in B- and T-cell lymphomas from these breeds by exome sequencing of tumor and normal pairs. Strong similarities were evident between B-cell lymphomas from golden retrievers and cocker spaniels, with recurrent mutations in *TRAF3-MAP3K14* (28% of all cases), *FBXW7* (25%), and *POT1* (17%). The *FBXW7* mutations recurrently occur in a specific codon; the corresponding codon is recurrently mutated in human cancer. In contrast, T-cell lymphomas from the predisposed breeds, boxers and golden retrievers, show little overlap in their mutation pattern, sharing only one of their 15 most recurrently mutated genes. Boxers, which develop aggressive T-cell lymphomas, are typically mutated in the PTEN-mTOR pathway. T-cell lymphomas in golden retrievers are often less aggressive, and their tumors typically showed mutations in genes involved in cellular metabolism. We identify genes with known involvement in human lymphoma and leukemia, genes implicated in other human cancers, as well as novel genes that could allow new therapeutic options.

Diffuse large B-cell lymphoma (DLBCL) is a genetically heterogeneous, aggressive form of non-Hodgkin lymphoma (NHL) and the most prevalent form of B-cell NHL in people. Approximately 30,000 DLBCL cases are diagnosed each year in the United States, of which only half are curable (Abramson and Shipp 2005). Molecular subclassifications of DLBCL are highly predictive of treatment outcome (Alizadeh et al. 2000). NHL can also arise from T cells, making up 10%–15% of all NHL cases. T-cell lymphomas are highly heterogeneous and several subtypes exist (Evens and Gartenhaus 2003; Iqbal et al. 2010).

Dogs have previously been proven useful in determining predisposing genetic markers for human diseases due to the breed structure caused by artificial breeding for phenotypic factors (Dodman et al. 2010; Wilbe et al. 2010; Shearin et al. 2012; Karlsson et al. 2013; Tang et al. 2014). A recent lymphoma study in dogs identified a gene (*TRAF3*) as being commonly mutated in both dog and human B-cell lymphomas (Bushell et al. 2015). However, this study did not separate canine tumors based on breed, preventing the discovery of somatic mutations reflecting genetic background. Since dog breeds represent genetic isolates, comparing dog breeds with differential predispositions to cancer can indicate the role of the genetic background and allow for better detection of mutations influenced by that genetic background. In dogs, malignant lymphoma is the most common tumor treated with chemotherapy, affecting dogs of all ages and breeds (Valli et al. 2013); however, the high rate of lymphoma in certain breeds and preferential cells of origin among different breeds indicates genetic risk factors (Modiano et al. 2005).

Approximately 70% of all canine lymphomas arise from B cells (Modiano et al. 2005; Ponce et al. 2010). The most common form of canine B-cell lymphoma is the clinical and histological equivalent of human DLBCL (Vail and MacEwen 2000; Modiano et al. 2005; Valli et al. 2011; Ito et al. 2014). As in humans, the CHOP-based chemotherapy protocols are the most effective treatment for canine B-cell lymphomas. T-cell lymphomas are less common in dogs, and the most aggressive types have a higher risk of relapse and early death (Ruslander et al. 1997; Dobson et al. 2001). Many T-cell lymphomas also have a low long-term survival frequency in humans.

Exome sequencing efforts in human lymphomas have shown that certain mutations are specific for lymphoma subtypes or shared between only a few subtypes (Zhang et al. 2014). For example, DLBCL typically have mutations affecting B-cell receptor signaling or subunits, e.g., *CD79A/B*, and in histone modifiers like *EZH2* and *MLL2*, *PRDM1*, *TP53*, *CARD11*, and *MYD88* (Lenz et al. 2008; Davis et al. 2010; Mandelbaum et al. 2010; Morin et al. 2010; Ngo et al. 2011; Lohr et al. 2012). *MYD88* is also recurrently mutated in other B-cell lymphomas like primary central nervous system lymphoma (PCNSL) (Gonzalez-Aguilar et al. 2012), and *MLL2* and *TP53* mutations have been reported for mantle cell lymphoma (MCL) (Zhang et al. 2014). The two main human DLBCL subtypes—activated B cell (ABC) and germinal center B cell (GCB)—which can be distinguished based on differential gene expression and prognosis (Alizadeh et al. 2000), also have unique recurrent mutations. ABC DLBCLs are characterized by mutations in B-cell differentiation genes and often show constitutive activation of NF-κB signaling, whereas *BCL2* and *MYC* translocations are characteristic of GCB DLBCLs (summarized in Pasqualucci et al. 2011b). Canine B-cell lymphomas can also be separated into germinal and post-germinal center types using gene expression data, sharing pathways with their human counterparts (Richards et al. 2013), although more studies are needed to fully elucidate this. T-cell lymphomas are much less studied compared with B-cell lymphomas, particularly DLBCL.

The treatment outcome of canine lymphoma is predictive of the human response to the same treatment (Honigberg et al. 2010; Marconato et al. 2013; London et al. 2014), and canine clinical trials, although highly regulated, are easier to complete compared with human trials. Hence, a better understanding of canine lymphoma offers great potential to accelerate development of new treatments for human patients. Here, we have compared the somatic mutations of B- and T-cell canine lymphoma in three dog breeds with different lymphoma immunophenotype predispositions. The identified breed-specific patterns are a good opportunity to study interventions targeting the significant mutations.

## Results

### General mutation load and significantly mutated genes

**Figure 1.**
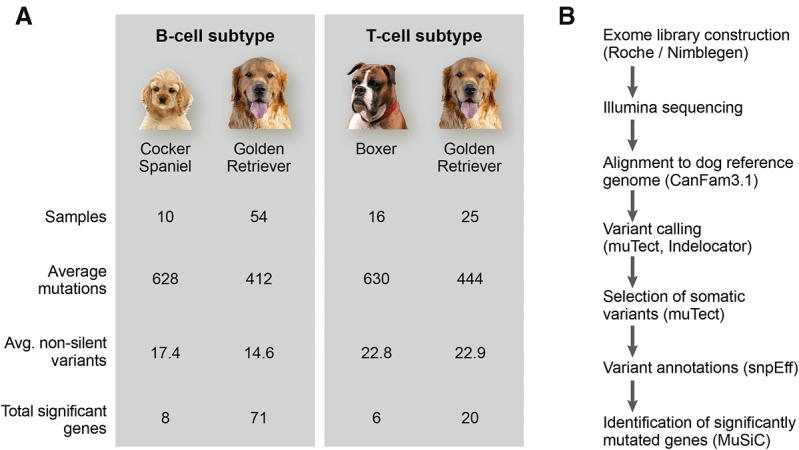
Analysis pipeline and mutation number overview. (*A*) Sample numbers and average mutations per breed and immunophenotype are indicated. (*B*) Exome libraries were sequenced and variants were called with MuTect, a caller adapted for cancer data, and IndeLocator. Somatic variants were annotated using SnpEff. MuSiC was used for identification of significantly mutated genes.

To allow detection of typical B- and T-cell lymphoma tumor mutations in different genetic backgrounds, samples were collected from three dog breeds predisposed to different lymphoma subtypes (Modiano et al. 2005). This is the largest canine lymphoma exome sequencing effort to date. The average lymphoma incidence has been estimated as 20–100 cases per 100,000 dogs (Ito et al. 2014). Golden retrievers in the United States are predisposed to both B- and T-cell lymphoma, with 13% developing lymphoma, and >60% developing cancer generally (Glickman et al. 2000). B- and T-cell lymphoma is equally common in this breed, indicating that they are relatively more predisposed to T-cell lymphoma, which normally makes up one-third of the lymphoma cases (Modiano et al. 2005). Boxers also show lymphoma predisposition (Modiano et al. 2005), and ∼90% of their lymphomas originate from T cells (Modiano et al. 2005; Pastor et al. 2009). For a breed typically developing B-cell lymphomas (relative frequency about 9:1 for B:T-cell), cocker spaniels were chosen (Modiano et al. 2005). The differential predisposition to lymphoma arising from different cell types reflects the homogeneous genetic backgrounds of dog breeds, different among breeds but relatively homogeneous within each breed. All three breeds chosen were created independently ∼200 yr ago, and are not related beyond the species level (Vaysse et al. 2011). The number of sequenced samples is shown in Figure 1; 64 B-cell lymphomas (54 from golden retriever [Gr B-cell] and 10 from cocker spaniel [Cs B-cell]) and 41 T-cell lymphomas (25 from golden retriever [Gr T-cell] and 16 from boxer [Bx T-cell]). The difference in sample group sizes reflects both the popularity of and the lymphoma frequency in each breed. The analysis pipeline is outlined in Figure 1. Matched tumor and normal samples were collected, and their exomes were captured via a Roche custom designed canine exome (50 Mb) followed by sequencing by Illumina (average depth normal 72×, tumor 93×). MuTect (Cibulskis et al. 2013) and IndeLocator (http://www.broadinstitute.org/cancer/cga/indelocator) were used to call variants and identify somatic mutations, which were then annotated with SnpEff (Cingolani et al. 2012). On average, 476 somatic mutations, of which 18 were nonsilent protein-coding mutations, were identified in each sample. T-cell lymphomas had a higher average load of nonsilent protein-coding mutations compared to B-cell lymphomas (23 versus 15 per sample, *P*_*t*-test_ = 2.3 × 10^−3^) (Fig. 1). The overall pattern of somatic mutations was similar across the breed and lymphoma immunophenotype groups (Supplemental Fig. S1), but golden retrievers have on average 33% fewer total mutations compared to the other two breeds (*p*_*t*-test_ = 2.8 × 10^−6^) (Fig. 1). The number of somatic mutations did not increase with sequencing depth (Supplemental Fig. S2), indicating that the sequencing was deep enough to comprehensively identify mutations and use these for comparison between breeds and tumor types.

**Figure 2.**
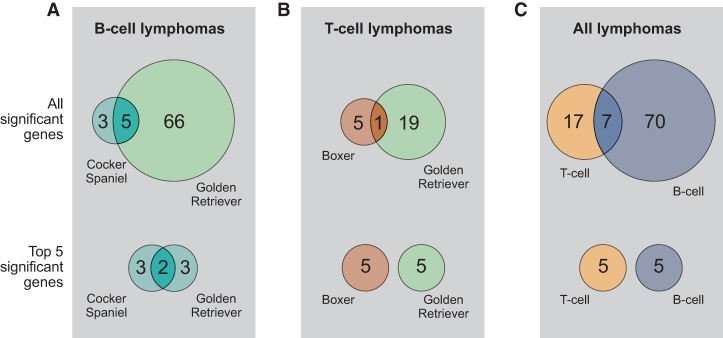
Overlap of significantly mutated genes. (*A*) The two B-cell lymphoma predisposed breeds share some of their most significantly mutated genes. (*B*) The two T-cell lymphoma predisposed breeds do not share any top significantly mutated genes. (*C*) There is some overlap in significantly mutated genes between the all B-cell lymphomas compared with the all T-cell lymphomas.

The nonsilent coding mutations, around 18 per sample (Fig. 1; Supplemental Table S1), were used to identify significantly mutated genes using the Genome MuSiC tool (Dees et al. 2012). The number of significantly mutated genes largely reflects the number of input samples. The vast majority (93%, 87 of 94) of significantly somatically mutated genes were significantly mutated in only one lymphoma immunophenotype group (Fig. 2). The top most significantly mutated genes in B-cell lymphomas are *POT1*, *FBXW7*, and *TRAF3* (Table 1; Supplemental Table S2). Other B-cell lymphoma defining genes include the *FAM90A1* uncharacterized protein, the tumor suppressor *TP53*, the RNA helicase *DDX3X*, proteasome subunit *PSMA1*, proline-rich nuclear receptor coactivator 1 (*PNRC1*), SET-domain containing 2 (*SETD2*), and mitogen-activated protein kinase kinase kinase 14 (*MAP3K14*). The large majority of those have high protein identity with their human counterparts (Supplemental Table S3A). Notably, *TRAF3* and *MAP3K14* (*NIK*) act in the same complex in the alternative NF-κB pathway. Both have been reported as mutated in human classical Hodgkin lymphoma (Otto et al. 2012) as well as mantle cell lymphoma, where MAP3K14 was recently reported as a new therapeutic target (Rahal et al. 2014). NF-κB pathway deregulation is also a feature of many DLBCL tumors (Compagno et al. 2009). *DDX3X* has been reported to be mutated in Burkitt's lymphoma (Richter et al. 2012). SETD2 is a histone methyltransferase. Several histone methyltransferases (primarily MLL2, EZH2) and acetyltransferases (typically CREBBP, EP300) have been implicated in human lymphoma (Morin et al. 2011; Pasqualucci et al. 2011a,b). As described for human DLBCL (Pasqualucci et al. 2011b), mutations in these genes are largely mutually exclusive among the studied B-cell lymphomas (Supplemental Fig. S3). In the T-cell lymphoma cohort, the most significantly mutated genes (Table 2) are SATB homeobox 1 (*SATB1*), followed by *TBC1D26* (uncharacterized protein), proteasome subunit *PSMA1*, cytochrome c oxidase subunit VIIIA (*COX8A*), and tumor suppressor *PTEN*. About half of the most significantly mutated genes in this study have been implicated in human lymphoma, but there are also several hits that have not previously been reported in human cancer studies, such as genes from the *NLRP* family, with a role in innate immunity. In addition, a smaller fraction of the significantly mutated genes, such as *PSMA1* and *KPNA2*, have not been reported in human lymphoma but have been reported in other human cancers.

**Table 1.**
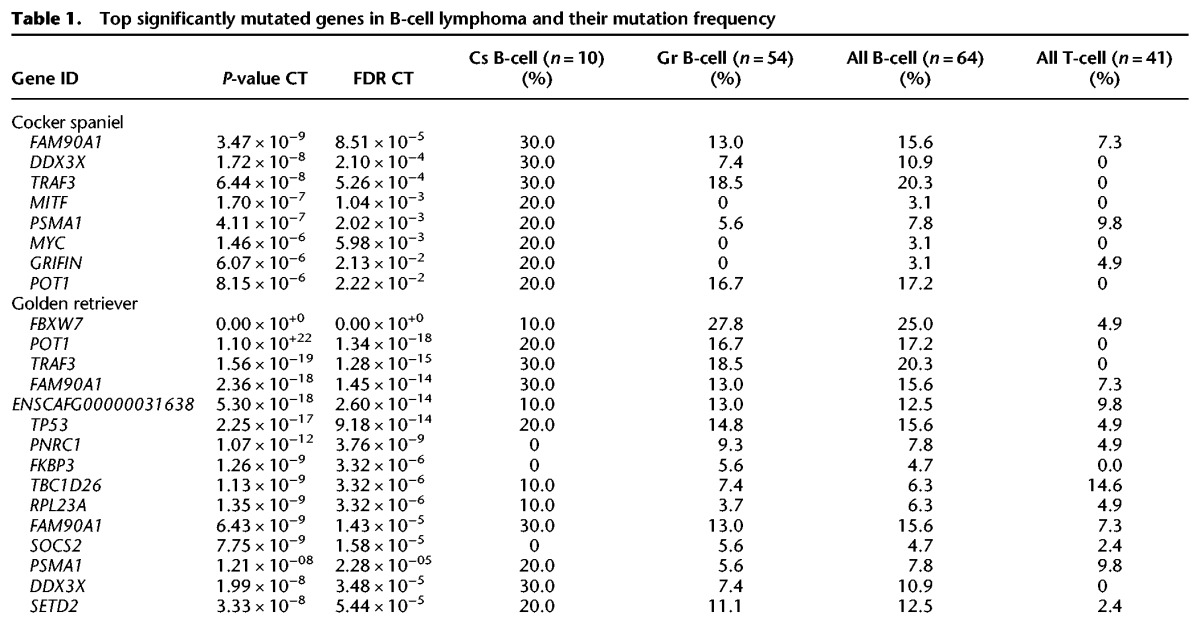
Top significantly mutated genes in B-cell lymphoma and their mutation frequency

**Table 2.**
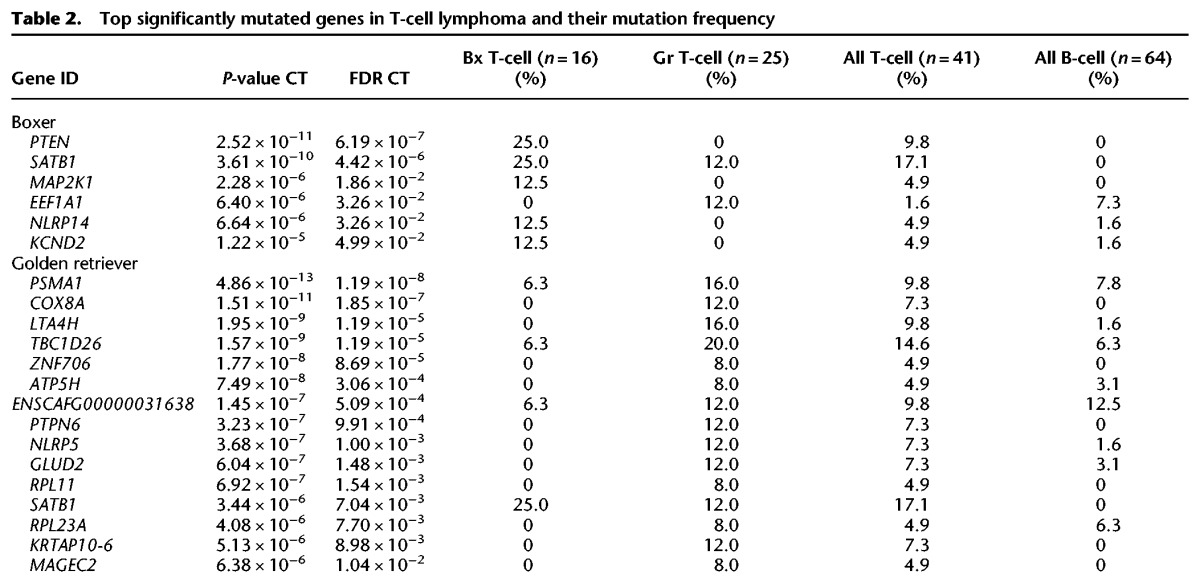
Top significantly mutated genes in T-cell lymphoma and their mutation frequency

### B-cell tumor mutations are more uniform than T-cell tumor mutations

Only seven genes (7%) were significantly (as determined by MuSiC analysis) mutated in both lymphoma immunophenotype groups, such as the proteasome subunit gene *PSMA1*, and the genes encoding the two uncharacterized proteins FAM90A1 and TBC1D26. With few exceptions, most genes are significantly mutated in either B- or T-cell lymphoma and not in both (Fig. 2; Supplemental Table S2).

B-cell lymphoma-defining genes show striking similarities between the two predisposed breeds. Of all significantly mutated genes in cocker spaniel B-cell lymphomas, 62.5% (5 of 8) are also among the 15 most significantly mutated golden retriever B-cell lymphoma genes (Fig. 2). Of the four top significant genes, two are shared. In contrast, among T-cell lymphomas, the significantly mutated genes are most often confined to only one breed (Table 2).

The effects of these mutations were estimated using Ingenuity Pathway Analysis (IPA; http://www.ingenuity.com) on the significantly mutated genes for each breed and tumor type. As the majority of significantly mutated canine genes have high protein identity with their human orthologs, pathways are expected to be conserved between canine and human. As expected from their differential sharing of typical mutations, the canonical pathways altered are highly overlapping between cocker spaniel and golden retriever B-cell lymphomas, and they are very different between boxer and golden retriever T-cell lymphomas. Seventy-five percent (34 of 46) of all canonical pathways altered by the genes that are significantly mutated in Cs B-cell lymphomas are also implicated for the Gr B-cell lymphoma tumors (Table 3; Supplemental Table S4). In contrast, only 15% of the Bx T-cell lymphoma canonical pathways are also implicated in Gr T-cell lymphoma tumors (Table 4; Supplemental Table S5). A similar pattern is seen when functional annotation charts generated from top significantly mutated genes using DAVID (Huang da et al. 2009a,b) are compared.

**Table 3.**
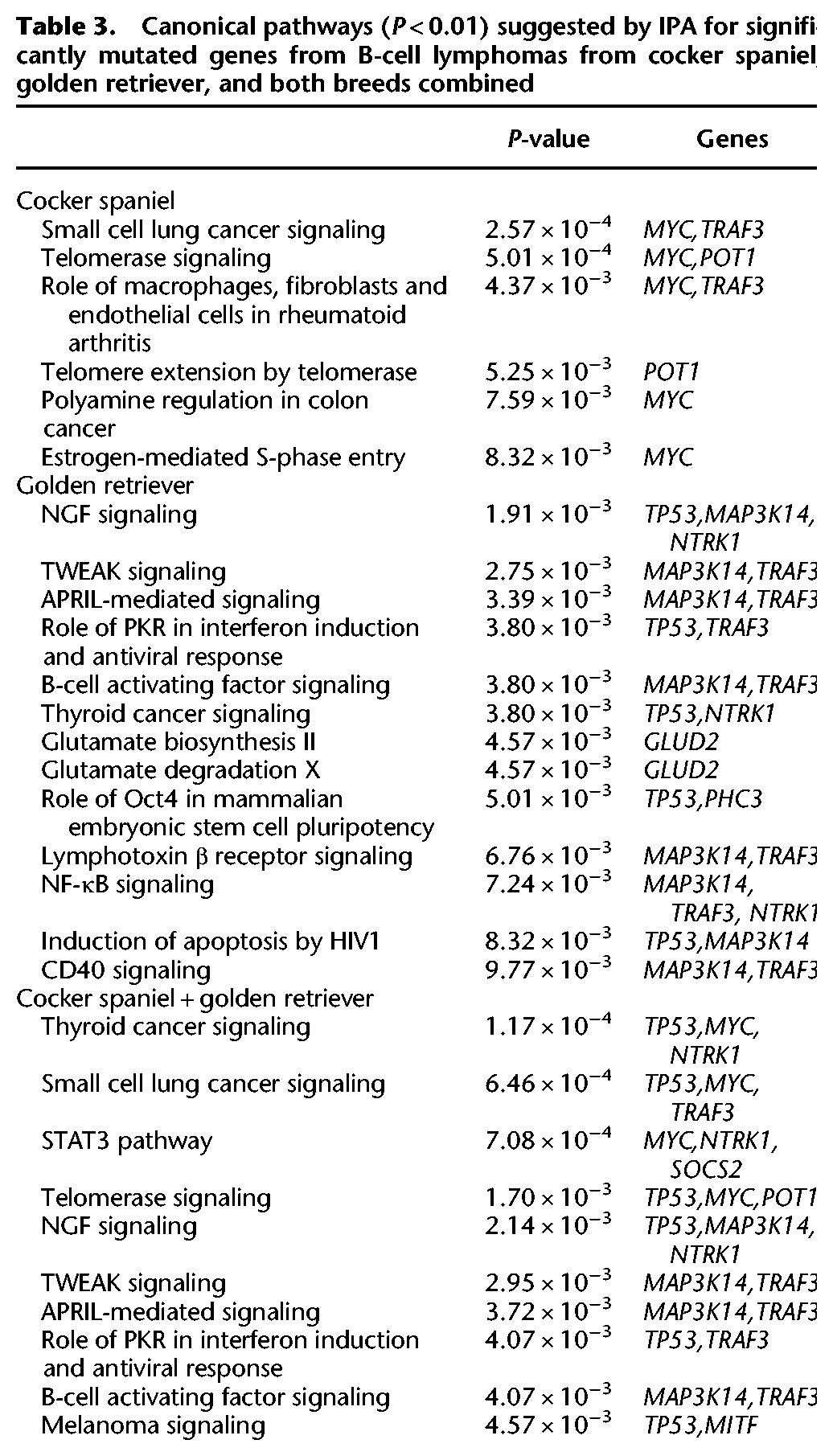
Canonical pathways (*P* < 0.01) suggested by IPA for significantly mutated genes from B-cell lymphomas from cocker spaniel, golden retriever, and both breeds combined

**Table 4.**
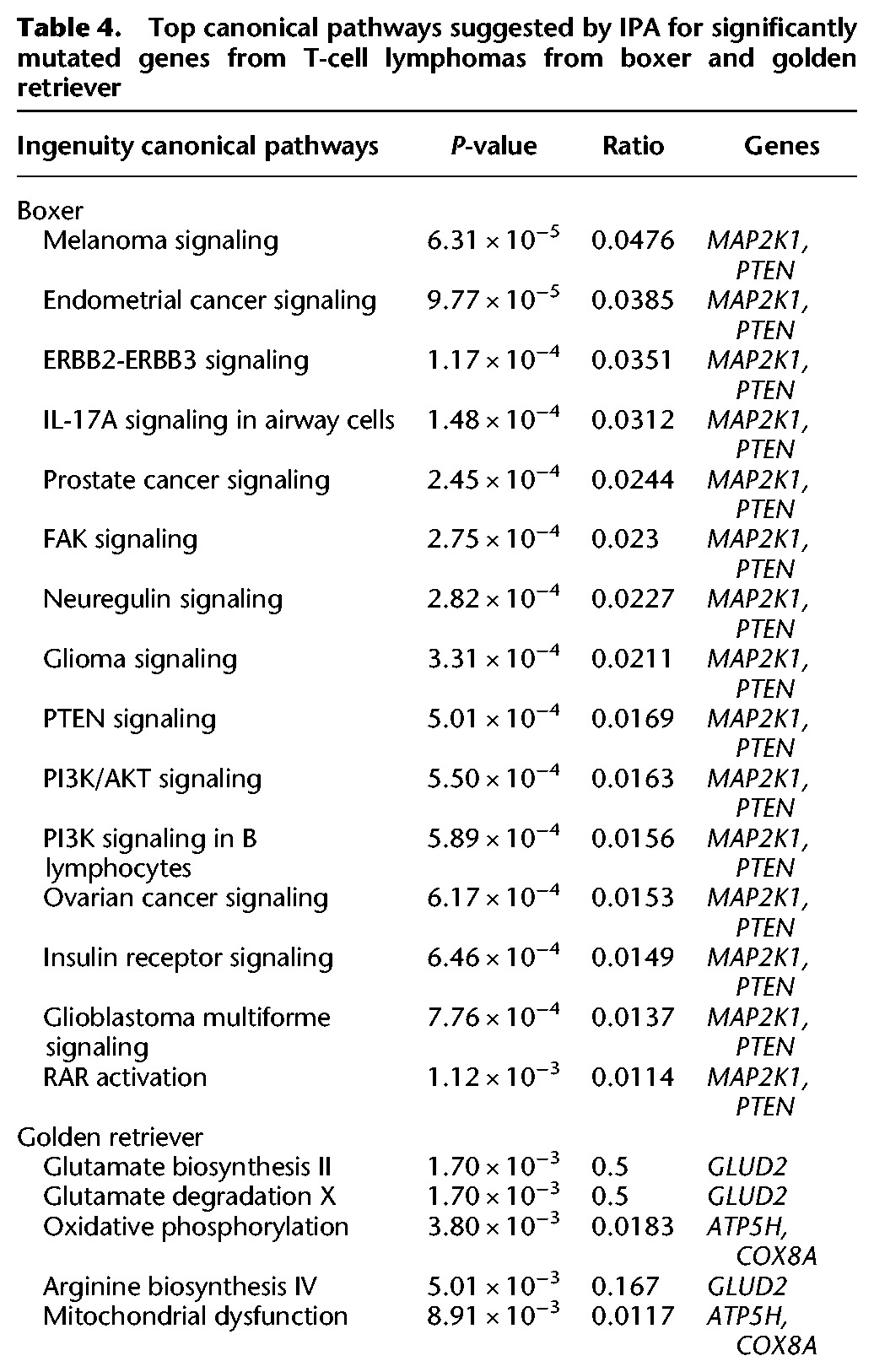
Top canonical pathways suggested by IPA for significantly mutated genes from T-cell lymphomas from boxer and golden retriever

Somatic copy number alterations (SCNAs) estimated from the exome sequencing data confirm the differences between B- and T-cell lymphomas. The most significant alterations are deletions in part of the T-cell receptor (*TCR*), which are typically seen in lymphomas originating from T cells (*P*_*t*-test_ = 1.7 × 10^−7^), and deletions in two immunoglobulin genes, more commonly seen in lymphomas originating from B cells (Table 5; Supplemental Table S6). These deletions are likely not a cancer-driven alteration, but rather *TCR* and immunoglobulin gene diversity reflecting the tumor cell of origin. In addition, the detected SCNAs (Supplemental Fig. S4) show overlap with a few of the recurrently mutated genes, such as *KLRK1*, a member of the killer cell lectin-like receptor subfamily K, and *PKD1*, which encodes a polycystin protein involved in cell–cell/matrix interactions and with a role in cell migration and invasion in human osteosarcoma (Onishi et al. 2012). The SCNAs are seen in both B- and T-cell lymphomas, although these two genes are only significantly mutated in B-cell lymphomas.

**Table 5.**
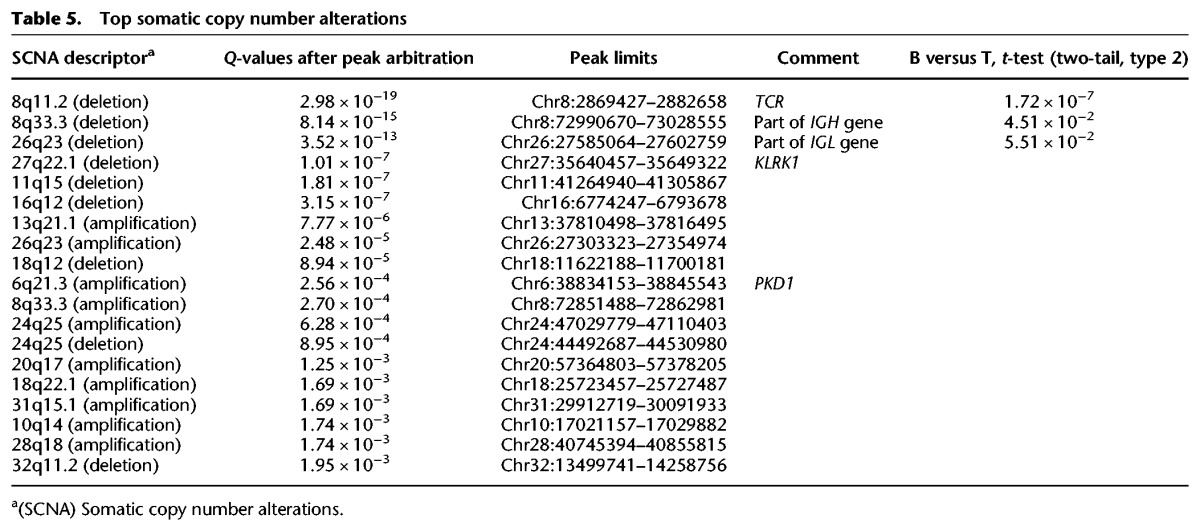
Top somatic copy number alterations

### B-cell lymphoma defining pathways point to the importance of telomerase and autoimmunity

The pathways affected by typical B-cell lymphoma tumor mutations highlight the importance of telomerase and proliferation signaling for successful tumor development, as well as pathways involved in autoimmunity. The top network estimated by IPA for the combined B-cell lymphoma data set is “Cancer, Hematological Disease, Immunological Disease” (Supplemental Table S7). The canonical pathways identified for the two B-cell lymphoma data sets, when analyzed separately as well as together, include pathways for specific cancers, telomerase signaling pathways, and pathways associated with proliferation/apoptosis and autoimmune disorders, such as TWEAK signaling, APRIL-mediated signaling, and the STAT3 pathway (Table 3; Supplemental Table S4). Cell death and immunity (CD40L and TNF receptor and ligand signaling) were also reported when the top significantly mutated genes were functionally annotated using DAVID (Huang da et al. 2009a,b) (Supplemental Table S8).

*POT1*, the most significantly somatically mutated gene in the Gr and Cs B-cell lymphomas combined, encodes a protein important for telomere maintenance, and mutations in this gene predispose to several types of cancer in human (Robles-Espinoza et al. 2014; Shi et al. 2014). The gene is also recurrently mutated, lost, or differentially regulated in human cancers, including lymphoma (Bellon et al. 2006; Vega et al. 2008).

**Figure 3.**
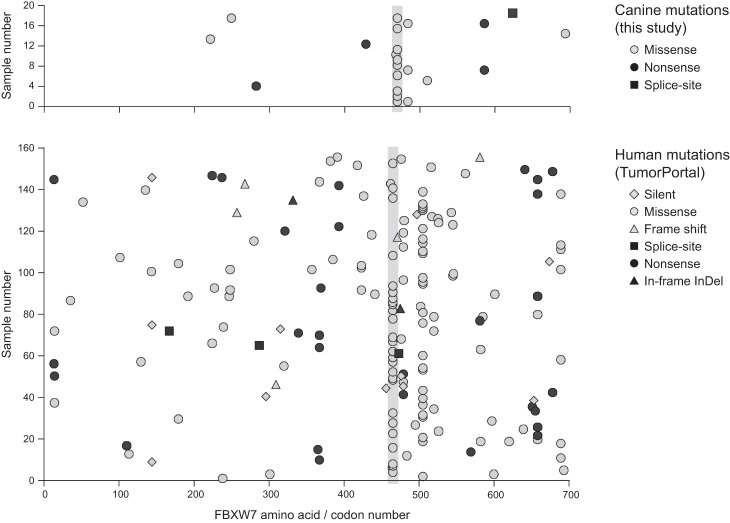
The same amino acid in FBXW7 is recurrently mutated in human and dogs. (*Upper*) Canine FBXW7 mutations show recurrent targeting of amino acid R470. (*Lower*) A subset of the mutations reported in various human cancers (TumorPortal) (Lawrence et al. 2014) shows recurrent mutations on two amino acid positions including R465, the equivalent of canine R470.

The second most significantly mutated gene among all B-cell lymphomas combined is the most commonly mutated gene among Gr B-cell lymphomas alone. This gene encodes the E3 ubiquitin ligase FBXW7, which targets cyclin E for degradation (Koepp et al. 2001) and controls the stability of MYC (Yada et al. 2004). FBXW7 has been shown to be recurrently mutated at two amino acid positions in human cancers (Lawrence et al. 2014). As shown in Figure 3, 41% of all *FBXW7* mutations in our lymphomas occur at the corresponding codon as one of those positions (canine R470, equivalent to human R465) (see Supplemental Table S3B). Notably, *FBXW7* is much more recurrently mutated in Gr B-cell than Cs B-cell lymphomas, and it is also mutated in a few Gr T-cell lymphomas but none of the studied Bx T-cell lymphomas.

The TNF-receptor associated factor TRAF3 is part of the CD40 signaling cascade regulating proliferation, immunoglobulin class switching, and apoptosis (Rousset et al. 1991; Tsubata et al. 1993; Kornbluth et al. 2012). TRAF3 also acts in a complex with TRAF2, BIRC2 (cIAP1), and BIRC3 (cIAP2) to target MAP3K14 (NIK) for degradation, thereby regulating the alternative NF-κB pathway regulating proliferation, B-cell activation, and other processes (for review, see Sun 2011). *TRAF3* alone is mutated in 30%, and either or both *TRAF3* and *MAP3K14* are mutated in 50% of the cocker spaniel B-cell lymphomas and 30% of all golden retriever B-cell lymphomas—those two genes are also commonly seen mutated in human classical Hodgkin's lymphoma (Table 1; Otto et al. 2012). A recent study, inspired by *TRAF3* mutations in canine B-cell lymphoma RNA-seq showed that *TRAF3* loss resulting in decreased gene expression is a feature of some human DLBCLs (Bushell et al. 2015). The *TRAF3* gene is also significantly mutated in multiple myeloma (Lawrence et al. 2014). *BIRC3* is mutated in mantle cell lymphoma and GCB DLBCL but not in Burkitt's lymphoma or ABC DLBCL (Zhang et al. 2014).

*FAM90A1*, the fourth most significantly mutated gene among B-cell lymphomas (16%) belongs to a gene family with 25 members in the human genome, several resulting from a primate-specific gene duplication (Bosch et al. 2007). Dogs have four paralogs, of which only one is recurrently mutated in B-cell lymphoma. The same paralog is mutated in 7% of the T-cell lymphomas. The clustering of the mutations in this gene suggests that they are functionally relevant. The exact function of this gene is unknown.

### Mutation patterns in T-cell lymphoma predisposed breeds overlap very little

In contrast to B-cell lymphomas, the T-cell lymphoma defining genes are largely not shared between the two predisposed breeds (golden retrievers and boxers). Of the 15 most commonly mutated genes in each of the two predisposed breeds, only one is shared (*SATB1*). Of the six significantly mutated genes identified in Bx T-cell lymphomas with MuSiC analysis, only one, *SATB1*, is reported mutated among the Gr T-cell lymphoma tumors (Table 2).

Since the significantly mutated genes in the two T-cell lymphoma breeds were very different, they were analyzed separately with IPA. The respective networks are not shared. Top networks are “Cellular Development, Cellular Growth and Proliferation, Cellular Movement” for Bx T-cell and “Connective Tissue Development and Function, Tissue Morphology, Cell-To-Cell Signaling and Interaction” for Gr T-cell lymphoma (Supplemental Table S9). Similarly, functional annotation charts generated using DAVID implicates cell adhesion and cell migration for the Boxer significantly mutated genes, whereas the gene ontology terms associated with the top significantly mutated genes from Gr T-cell lymphomas mostly revolve around ribosomes and mitochondria. The canonical pathway suggested to be affected for Bx T-cell lymphomas reflect the recurrent mutations in *PTEN* and *MAP2K1*; these two genes are mutated in 38% of all Bx T-cell lymphomas and not in a single Gr T-cell lymphoma or any of the B-cell lymphomas in this study (Table 2).

Bx T-cell lymphomas were typically mutated in *SATB1* and *PTEN*. SATB1 is a matrix protein recruiting chromatin remodeling factors, thereby regulating chromatin state and gene expression. This gene was mutated in 25% of all Bx and 12% of the Gr T-cell lymphomas. The well-known tumor suppressor gene *PTEN* is mutated in 25% of all Bx and none of the Gr T-cell lymphomas. Several human cancers show deregulation of the PI3K pathway (Samuels et al. 2004). The *PI3KCD*, *PIK3RI*, and *MTOR* genes have been reported to be mutated in occasional DLBCL cases (Zhang et al. 2013). PTEN antagonizes the PI3K-AKT-mTOR pathway (Maehama and Dixon 1998; for review, see Song et al. 2012; Worby and Dixon 2014), and at least one of those four genes are mutated in almost half (44%) of all Bx T-cell lymphomas but only in 6% of all Gr T-cell lymphomas (Table 2), suggesting that this pathway is highly important for Bx T-cell lymphoma development. Hypothesizing that it may be something in the genetic background of boxers that increases their likelihood of getting mutations in the PTEN-mTOR pathway, other important genes connected to this pathway were evaluated for germline mutations in exome-sequenced blood DNA from boxers and non-boxers, identifying a nonsynonymous variant in *FOS*. Eighty-one percent of the boxers (*n* = 16) are heterozygous or homozygous for the variant, compared to 4% of the non-boxers (*n* = 210). The variant, on canine Chr 8: 48333412, changes a serine to an isoleucine and is predicted to be “probably damaging” by PolyPhen-2 (Adzhubei et al. 2010). It is, however, impossible to say if this variant is linked to the detected mutation frequency in the PTEN-mTOR pathway in boxers without further analysis.

The most significantly mutated genes in Gr T-cell lymphomas are *PSMA1*, the cytochrome C oxidase subunit *COX8A*, and leukotriene A4 hydrolase (*LTA4H*). *LTA4H* and *PSMA1* have not been associated with human T-cell lymphoma, but have been associated with other human cancers (Chen et al. 2003; Onken et al. 2010; Jansen et al. 2012) and indicate new treatment options for Gr T-cell lymphoma. Proteasome subunit gene *PSMA1* is significantly mutated in both B- and T-cell lymphomas. COX8A is a cytochrome c oxidase subunit, which is involved in apoptosis (Liu et al. 1996). Inhibition of LTA4H is associated with chronic inflammation (Snelgrove et al. 2010; Wells et al. 2014), and mutations in this gene may reflect de-differentiation of the tumor, although the recurrent nature of the mutations rather suggests a gain-of-function mutation.

T-cell lymphoma tumors from both boxers and golden retrievers also have significant mutations in separate members of the *NLRP* gene family. Those two members have not previously been implicated in cancer. *NLRP5* is significantly mutated in golden retriever lymphomas, and *NLRP14* is significantly mutated in boxer lymphomas (Table 2).

## Discussion

Breeding to select for phenotypic traits in dogs has created genetic isolates (breeds) that not only present with wide morphologic variation, but also have developed differential predispositions to disease. Using different breeds, we see that the two breeds predisposed to B-cell lymphoma have large overlaps in commonly mutated genes and pathways, whereas T-cell lymphomas from two predisposed breeds are very different. There is no population structure between those three breeds. The breeds diverged from the ancestral dog population independently and at essentially the same time (Vaysse et al. 2011), so the similarity between B-cell lymphomas cannot be explained by higher relatedness between the predisposed breeds. In particular, the B-cell lymphomas are strikingly similar to human B-cell lymphomas. We see recurrent mutations affecting NF-κB signaling and histone modifiers, both of which are typically seen in human DLBCL. The two T-cell lymphoma breeds typically develop different types of T-cell tumors, and their mutation patterns are more different. This raises the possibility that genetic background predisposes to different subtypes of T-cell lymphoma and may help dissect the genetic underpinnings of different T-cell types. It is therefore important to understand which human lymphoma subtypes the lymphomas in different breeds are most similar to.

The difference in mutation pattern between T-cell lymphomas from boxers and golden retrievers is particularly interesting. Boxers often develop lymphoblastic T-cell lymphomas as well as peripheral T-cell lymphoma (PTCL) (Lurie et al. 2008), both aggressive forms of T-cell lymphoma (Frantz et al. 2013), whereas golden retrievers are more prone to developing T-zone lymphomas that are relatively mild and have not been described in humans (Fosmire et al. 2007; Seelig et al. 2014). The typical mutation pattern in those two breeds is remarkably distinct. Due to the number of different subtypes present in each breed and the number of individuals without subtype information available in this study, it is impossible to say whether the genetic differences between subtypes are smaller or larger than the genetic difference between tumors from different breeds (Supplemental Material). Further studies are required to determine if somatic mutations reflect the subtype, which has a genetic predisposing component, or if somatic mutations directly reflect genetic background, or a combination thereof. The mutations in PTEN-mTOR pathway in half (44%) of all Bx T-cell lymphomas suggest that this pathway is highly important for the formation of aggressive lymphomas. In human cancers, *PTEN* is most commonly lost in endometrial cancer and glioblastoma (Lawrence et al. 2014). Uterine cancers are generally uncommon among all US dogs due to spaying practices, but boxers have an increased frequency of glioma. It can be speculated that boxers may be “predisposed” for a mutation in this pathway—that is, that their genetic background gives them an increased sensitivity for such mutations. Interestingly, 81% of all the boxers sequenced here carry a germline variant in *FOS*. Only 4% of the exome-sequenced non-boxer dogs (*n* = 210) carry this variant. As the FOS transcription factor controls some of the same downstream targets of the PTEN-mTOR pathway (Koul et al. 2007; De Marco et al. 2013), it is possible that the genetic background of a boxer could make them more sensitive to a mutation in the PTEN-mTOR pathway in relevant cell types compared to the average dog. It has been shown that genetic background predisposes to specific cancers, even specific lymphoma subtypes. This could happen through the genetic background making the individual more sensitive to mutations in specific cancer genes that determine disease subtype. Genetic background also affects overall tumor mutation load, as evident from the lower somatic mutation frequency in golden retrievers compared with the two other breeds.

The B-cell lymphomas may also be subdivided based on their mutation profile. *TRAF3-TRAF2-BIRC2-BIRC3-MAP3K14* are mutated in 30% of all B-cell lymphomas, and 50% of all Cs B-cell lymphomas, which is about the same fraction as human DLBCL tumors with dysregulated canonical or noncanonical NF-κB pathways (Compagno et al. 2009). In contrast to the T-cell lymphomas, we see very few differences in significantly mutated genes between B-cell lymphomas for the two studied breeds. This certainly reflects a more uniform mutation pattern, but it is possible that there are subtle differences between the two breeds that we do not pick up because of the relatively low number of cocker spaniel samples included in this study. For example, *FBXW7* appears to be more recurrently mutated in B-cell lymphomas from golden retriever compared to cocker spaniels, but this difference is not statistically significant.

The recurrent mutations in specific genes and pathways are likely reflecting the relatively homogeneous genetic background of each breed, allowing us to identify molecular subtypes based on genetic background. Such genetic backgrounds may be exemplified by the two loci predisposing to B-cell lymphoma and hemangiosarcoma in golden retrievers (found by genome-wide association mapping), which are associated with differential expression of immune-related genes, including *BIRC3* (Tonomura et al. 2015). These results point to a mechanism involving altered T-cell activation as a strong predisposing factor in this breed. All three breeds analyzed here for lymphoma tumor mutation also have an increased incidence of autoimmune disorders like atopic dermatitis. The risk of developing several lymphoma subtypes, including DLBCL, is elevated in patients with autoimmune diseases like Sjögren's syndrome and SLE (lupus) (Smedby et al. 2006). This could suggest that the lymphoma predisposition in the breeds comes from an unbalanced immune regulation.

Several of the recurrently mutated genes have not previously been implicated in human lymphoma, including NLRP5, NLRP14, and GRIFIN. This follows the pattern reported previously in human studies, suggesting that the majority of mutations specific to certain cancer subtypes or relatively rare mutations have not yet been reported (Lawrence et al. 2014). Those novel mutations, and mutations reported in other cancers but not lymphoma, may indicate novel treatment possibilities in lymphoma. Generally, the same chemotherapeutic agents are effective in both dogs and humans (Honigberg et al. 2010; London et al. 2014). Hence, it is highly likely that the significantly mutated genes identified in dogs are suggestive of genes and pathways involved in human lymphoma, possibly allowing new treatment options. Several of the most significantly mutated genes in dogs, such as *MITF*, *FKBP3*, and *LTA4H*, have been identified in a small proportion of human lymphoma cases, suggesting that the homogeneous genetic background of dog breeds may highlight mutations important for, and possibly guiding treatment for, a subset of human patients.

Looking at the most significantly mutated genes in B-cell and T-cell lymphomas across the three studied breeds, genes not previously implicated in human lymphoma include well-known cancer-associated genes such as *LTA4H* and *PSMA1*, indicating potential treatment with proteasome inhibitors. In contrast, *TBC1D26* is a novel cancer gene, which may allow for completely new treatments. *TBC1D26* has not been well characterized, but belongs to a family of proteins in which other members have been implicated in cell cycle control. *TBC1D26* paralogs are mutated in 17% of all samples, with the *ENSCAFG00000025100* paralog being the most commonly mutated, and the mutations cluster in a few domains. Additionally, each T-cell lymphoma predisposed breed is significantly mutated in a different member of the *NLRP* gene family, which is primarily associated with a role in innate immunity (Pedraza-Alva et al. 2015) as well as reproduction (Duéñez-Guzmán and Haig 2014).

Canine studies may be more translational at the pathway level than at the gene level for example histone acetyltransferases and methyltransferases are recurrently mutated in human lymphomas; and although the exact genes only partially overlap, other histone modifiers are mutated in the dog. This affirms the observation that the mechanisms of lymphoma overlap strongly between humans and dogs.

In conclusion, our results highlight how the spontaneously occurring lymphomas in purebred dogs can be very helpful in characterizing different tumor molecular subtypes, which should contribute to the understanding of both canine and human lymphomas.

## Methods

### Sample collection

Tissue samples (lymph node with tumor and matched blood or healthy lymph node) were provided by coauthors, veterinary hospitals, or obtained from the Pfizer Canine Comparative Oncology and Genomics Consortium (CCOGC) Biospecimen Repository. Investigators at other institutions may have received specimens from the same subjects from CCOGC. Naïve (newly diagnosed and untreated) disease was one of the eligibility criteria for participation. For lymph node details, see Supplemental Material.

### Immunophenotyping

Immunophenotyping information was supplied with the samples, or determined by immunohistochemistry using CD3/CD79A markers, or by PARR (Burnett et al. 2003). Subtyping information (DLBCL, TZL, PTCL, etc.) was only available for one-third of the samples and hence not considered for the analysis (see Supplemental Material).

### Library construction and sequencing

Dog exome libraries were generated from standard indexed Illumina libraries using a custom Roche/Nimblegen solution-based capture library (120705_CF3_Uppsala_Broad_EZ_HX1) following the protocol provided. The libraries capture 85% of the canine exonic regions. Per suggestion from Nimblegen, developer's reagent (06684335001) was used in place of COT-1. Index-specific hybridization enhancing oligos were used.

### Alignment and filtering

After capture, tumor and normal indexed libraries were pooled separately into groups of eight. All pools were run on an Illumina HiSeq 2000 platform, with coverage targets of 30× for normal pools and 60× for tumor pools.

The reads were aligned to the CanFam3.1 reference genome (Hoeppner et al. 2014) using BWA 0.5.9 (Li and Durbin 2009). Using the Picard tool kit (http://broadinstitute.github.io/picard/), the duplicate reads were marked, and the data were sorted. Using the Genome Analysis Toolkit (GATK) software, local realignments were generated with cocleaning of tumor/normal sample pairs, and base quality scores were recalibrated per GATK Best Practices (Van der Auwera et al. 2013). Samples with fewer than 50% of target bases covered to 10× were removed.

### Variant calling

GATK 2.6 (DePristo et al. 2011) was used to realign the reads around indels. MuTect 1.1.4 (http://www.broadinstitute.org/cancer/cga/mutect) and IndeLocator (http://www.broadinstitute.org/cancer/cga/indelocator) were used to call SNPs and indels, respectively, on the resulting BAM files. Samples with more than 5 SD more variants than the mean were removed since this may reflect incorrect pairing of tumor and normal DNA. MuTect uses a statistical model to determine the likelihoods based on the reads of a position being at least partially nonreference in tumor and of being homozygous reference in normal. If these likelihoods are high enough, it concludes that a somatic event has happened. Variants considered by these programs to be somatic rather than germline were retained, and SnpEff 3.3 (Cingolani et al. 2012) was used to predict the possible effects of these variants. Although SnpEff reports all possible effects of a particular variant, we retained only one effect for each variant, choosing that which was judged to be most impactful. The resulting VCF files were compiled into a single MAF file using custom code.

### Variant filtering

The somatic variants were filtered to remove false positives due to germline population variation. This was done in two steps: First, previously known population variation was removed from the somatic set (Lindblad-Toh et al. 2005; Vaysse et al. 2011; Axelsson et al. 2013) and supplemented with additional variants identified by Erik Axelsson (E Axelsson and K Lindblad-Toh, unpubl.). Second, variants were removed that were found to be present in a panel of normal canine samples. Variants in these normal samples were called using SAMtools 1.1 and BCFtools 1.1 (http://www.htslib.org/). Normal variants were considered “true” if the Phred-scaled likelihood score of being homozygous reference at that position was greater than 50, meaning, if it was sufficiently likely that at least one allele was variant. The variant was “trusted” if the same variant was true in two or more samples. Indels could additionally be considered trusted if two or more indels were found to be true at that position (including the indel in question). All trusted variants were filtered out of the MuTect output files. Subsequently, putatively somatic variants within immunoglobulin genes and T-cell receptors (where variation is likely not reflecting the tumor) and those in olfactory receptors were removed. Finally, the top 25 significantly mutated genes from each breed group were scored for recurrent mutations. These were evaluated manually in IGV (Thorvaldsdóttir et al. 2012) and removed if the variant was considered highly likely to be a false positive, for example, because of read alignment problems due to insufficient cocleaning or if the vast majority of variants were supported by reads whose mate aligned to another chromosome with strong similarity to this region.

### Significantly mutated genes

The filtered mutation file, along with the original BAM files and Ensembl's dog annotation v.75 (Hoeppner et al. 2014) were run through Genome MuSiC 0.4 (Dees et al. 2012) to produce a list of significantly mutated genes. Default settings were used, except if multiple mutations were seen in a gene in one individual, they were counted as one (“merge concurrent” option). Multiple mutations in a gene could reflect mutations in different subclones, mutations in both gene copies in one cell, or several mutations targeting function, but it could also arise from lower purifying selective pressure on a gene whose function was already lost.

### Pathway analysis

Ingenuity pathway analysis (http://www.ingenuity.com) was used for pathway analysis using standard settings. Human gene names and MuSiC CT *P*-values were used. For analysis using DAVID (Huang da et al. 2009a,b), the top 15 (or all, if less than 15) significantly mutated genes were inputed as “gene list.”

### SCNA

Somatic copy number alterations were estimated from exome data by adapting the SegSeq algorithm (Chiang et al. 2009). Only 40 dogs could be used due to a recurrent artifact pattern, hence, all samples were analyzed together. DNA segments subject to somatic copy number alterations were analyzed with GISTIC 2.0 (Mermel et al. 2011).

## Data access

The sequence data from this study have been submitted to the NCBI Sequence Read Archive (SRA; http://www.ncbi.nlm.nih.gov/sra/). Accession numbers are listed in Supplemental Table S10. Somatic and germline variants have been deposited in NCBI dbSNP (http://www.ncbi.nlm.nih.gov/SNP/) under the assay IDs (ss#) listed in the Supplemental Material, and have been integrated into the “Broad Improved Canine Annotation v1” track hub at the UCSC Genome Browser (http://genome.ucsc.edu/cgi-bin/hgHubConnect). The tracks are called “Lymphoma som SNPs” and “Germline PON SNPs,” respectively.

## Competing interest statement

A patent (BI-2014/089 - B1195.70034US00) has been filed.

## Supplementary Material

Supplemental Material
