# Supplementary material for: Exome sequencing of lymphomas from three dog breeds reveals somatic mutation patterns reflecting genetic background
Source: Genome Res. 2015 Nov;25(11):1634–45. doi: 10.1101/gr.194449.115 (PMC4617960; doi:10.1101/gr.194449.115)
Supplement: Supplemental Material [file supp_25_11_1634__index.html]

Exome sequencing of lymphomas from three dog breeds reveals somatic mutation patterns reflecting genetic background — Supplemental Material 

# Exome sequencing of lymphomas from three dog breeds reveals somatic mutation patterns reflecting genetic background

## Supplemental Material

**Files in this Data Supplement:**

- Supp Table1.pdf
- Supp Table 10.pdf
- Supp Table 2.pdf
- Supp Table 3.pdf
- Supp Table 4.pdf
- Supp Table 5.pdf
- Supp Table 6.pdf
- Supp Table 7.pdf
- Supp Table 8.pdf
- Supp Table 9.pdf
- Supplemental Figures.docx
- Supplemental Information.docx
- Supp Germline variants ssIDs.txt.gz
- Supp Somatic variants ssIDs.txt.gz
