# Supplementary material for: Exome sequencing of lymphomas from three dog breeds reveals somatic mutation patterns reflecting genetic background
Source: Genome Res. 2015 Nov;25(11):1634–45. doi: 10.1101/gr.194449.115 (PMC4617960; doi:10.1101/gr.194449.115)
Supplement: Supplemental Material [file supp_gr.194449.115_Supp_Table1.pdf]

Supplementary Table 1. Number of tumor-normal paired samples analyzed, and average number of total and non-synonymous mutations per sample

		Number of samples	Total mutations per sample, average	NS coding mutations per sample, average	
B-cell	Cocker spaniel	10	628	17.4	
	Golden retriever	54	412	14.6	
T-cell	Boxer	16	630	22.8	
	Golden retriever	25	444	22.9	
all	all B	64	447	15.0	p (B vs T) = 4.3x10-3
	all T	41	521	22.8	
	all Golden retriever	79	422		p (Gr vs Bx+Cs) = 2.8x10-6
	all Boxer + Cocker spaniel	35	629		
	total	105	476	18.1	
