# Supplementary material for: Exome sequencing of lymphomas from three dog breeds reveals somatic mutation patterns reflecting genetic background
Source: Genome Res. 2015 Nov;25(11):1634–45. doi: 10.1101/gr.194449.115 (PMC4617960; doi:10.1101/gr.194449.115)
Supplement: Supplemental Material [file supp_gr.194449.115_Supp_Table10.pdf]

Supplementary Table 10. SRS and BioSample IDs for all samples analyzed

Sample ID	SRS ID	BioSample ID	Breed	Immuno-phenotype or Normal
CCB010028 0200	SRS878735	SAMN03436471	Boxer	Normal
CCB010028 0102	SRS879021	SAMN03436679	Boxer	T-cell
CCB020030 0200	SRS878863	SAMN03436531	Boxer	Normal
CCB020030 0100	SRS879443	SAMN03436936	Boxer	T-cell
CCB030166 0200	SRS878857	SAMN03436525	Boxer	Normal
CCB030166 0103	SRS878779	SAMN03436502	Boxer	T-cell
CCB030212 0201	SRS879207	SAMN03436852	Boxer	Normal
CCB030212 0100	SRS878476	SAMN03436216	Boxer	T-cell
CCB030292 0201	SRS878948	SAMN03436607	Boxer	Normal
CCB030292 0101	SRS879120	SAMN03436780	Boxer	T-cell
CCB040265 0200	SRS878874	SAMN03436542	Boxer	Normal
CCB040265 0102	SRS878699	SAMN03436295	Boxer	T-cell
CCB060027 0201	SRS878850	SAMN03436518	Boxer	Normal
CCB060027 0100	SRS878855	SAMN03436523	Boxer	T-cell
CCB060032 0201	SRS878875	SAMN03436543	Boxer	Normal
CCB060032 0102	SRS878892	SAMN03436559	Boxer	T-cell
CCB060034 0201	SRS879397	SAMN03436890	Boxer	Normal
CCB060034 0103	SRS878936	SAMN03436603	Boxer	T-cell
CasLea_Normal	SRS879202	SAMN03436847	Boxer	Normal
CasLea_Tumor	SRS878584	SAMN03436268	Boxer	T-cell
MurVoe_Normal	SRS879191	SAMN03436832	Boxer	Normal
MurVoe_Tumor	SRS878480	SAMN03436220	Boxer	T-cell
NygRoy_Normal	SRS879035	SAMN03436692	Boxer	Normal
NygRoy_Tumor	SRS878896	SAMN03436563	Boxer	T-cell
RoxRho_Normal	SRS879003	SAMN03436661	Boxer	Normal
RoxRho_Tumor	SRS879077	SAMN03436718	Boxer	T-cell
SopCas_Normal	SRS878842	SAMN03436511	Boxer	Normal
SopCas_Tumor	SRS878701	SAMN03436297	Boxer	T-cell
SopDix_Normal	SRS878724	SAMN03436460	Boxer	Normal
SopDix_Tumor	SRS878657	SAMN03436277	Boxer	T-cell
SteHel_Normal	SRS879139	SAMN03436807	Boxer	Normal
SteHel_Tumor	SRS878897	SAMN03436564	Boxer	T-cell
CCB010054 0203	SRS879113	SAMN03436773	Cocker spaniel	Normal
CCB010054 0107	SRS879126	SAMN03436785	Cocker spaniel	B-cell
CCB010099 0200	SRS879107	SAMN03436732	Cocker spaniel	Normal
CCB010099 0104	SRS878866	SAMN03436534	Cocker spaniel	B-cell
CCB030021 0200	SRS879442	SAMN03436935	Cocker spaniel	Normal
CCB030021 0100	SRS879017	SAMN03436675	Cocker spaniel	B-cell
CCB030193 0200	SRS878642	SAMN03436273	Cocker spaniel	Normal
CCB030193 0104	SRS879039	SAMN03436696	Cocker spaniel	B-cell
CCB040180 0201	SRS878719	SAMN03436455	Cocker spaniel	Normal
CCB040180 0101	SRS878839	SAMN03436508	Cocker spaniel	B-cell
CCB060135 0201	SRS878740	SAMN03436476	Cocker spaniel	Normal

CCB060135 0104	SRS878664	SAMN03436284	Cocker spaniel	B-cell
CCB070086 0201	SRS878868	SAMN03436536	Cocker spaniel	Normal
CCB070086 0100	SRS878877	SAMN03436545	Cocker spaniel	B-cell
CCB070124 0200	SRS878870	SAMN03436538	Cocker spaniel	Normal
CCB070124 0101	SRS878893	SAMN03436560	Cocker spaniel	B-cell
CCB070131 0200	SRS878660	SAMN03436280	Cocker spaniel	Normal
CCB070131 0103	SRS879095	SAMN03436721	Cocker spaniel	B-cell
CCB070289 0200	SRS879438	SAMN03436931	Cocker spaniel	Normal
CCB070289 0103	SRS879006	SAMN03436664	Cocker spaniel	B-cell
CCB010009 200	SRS878970	SAMN03436628	Golden retriever	Normal
CCB010009 101	SRS878729	SAMN03436465	Golden retriever	B-cell
CCB010010 200	SRS878956	SAMN03436614	Golden retriever	Normal
CCB010010 103	SRS878903	SAMN03436570	Golden retriever	B-cell
CCB010036 200	SRS879168	SAMN03436809	Golden retriever	Normal
CCB010036 104	SRS878539	SAMN03436266	Golden retriever	B-cell
CCB010040 200	SRS879231	SAMN03436864	Golden retriever	Normal
CCB010040 103	SRS878654	SAMN03436274	Golden retriever	B-cell
CCB010153 201	SRS878907	SAMN03436574	Golden retriever	Normal
CCB010153 100	SRS879010	SAMN03436668	Golden retriever	B-cell
CCB010182 201	SRS879170	SAMN03436811	Golden retriever	Normal
CCB010182 103	SRS878985	SAMN03436643	Golden retriever	B-cell
CCB010214 200	SRS878886	SAMN03436554	Golden retriever	Normal
CCB010214 103	SRS878659	SAMN03436279	Golden retriever	B-cell
CCB010307 201	SRS879394	SAMN03436888	Golden retriever	Normal
CCB010307 103	SRS879097	SAMN03436723	Golden retriever	B-cell
CCB010346 200	SRS879068	SAMN03436711	Golden retriever	Normal
CCB010346 104	SRS878718	SAMN03436454	Golden retriever	B-cell
CCB020009 200	SRS879065	SAMN03436708	Golden retriever	Normal
CCB020009 101	SRS879195	SAMN03436836	Golden retriever	B-cell
CCB020104 200	SRS878965	SAMN03436623	Golden retriever	Normal
CCB020104 102	SRS878929	SAMN03436596	Golden retriever	B-cell
CCB020249 200	SRS879198	SAMN03436839	Golden retriever	Normal
CCB020249 100	SRS879031	SAMN03436688	Golden retriever	B-cell
CCB030117 201	SRS879033	SAMN03436690	Golden retriever	Normal
CCB030117 103	SRS879110	SAMN03436744	Golden retriever	B-cell
CCB030130 200	SRS878720	SAMN03436456	Golden retriever	Normal
CCB030130 102	SRS878847	SAMN03436515	Golden retriever	B-cell
CCB030145 201	SRS879112	SAMN03436772	Golden retriever	Normal
CCB030145 104	SRS879127	SAMN03436786	Golden retriever	B-cell
CCB030251 200	SRS878768	SAMN03436495	Golden retriever	Normal
CCB030251 103	SRS878934	SAMN03436601	Golden retriever	B-cell
CCB030268 201	SRS879096	SAMN03436722	Golden retriever	Normal
CCB030268 103	SRS879173	SAMN03436814	Golden retriever	B-cell
CCB030346 201	SRS879116	SAMN03436776	Golden retriever	Normal
CCB030346 102	SRS878924	SAMN03436591	Golden retriever	B-cell
CCB030362 200	SRS879431	SAMN03436924	Golden retriever	Normal

CCB030362 103	SRS878667	SAMN03436287	Golden retriever	B-cell
CCB040038 201	SRS878912	SAMN03436579	Golden retriever	Normal
CCB040038 101	SRS879179	SAMN03436819	Golden retriever	B-cell
CCB040143 200	SRS879176	SAMN03436816	Golden retriever	Normal
CCB040143 104	SRS879069	SAMN03436712	Golden retriever	B-cell
CCB040222 200	SRS879188	SAMN03436829	Golden retriever	Normal
CCB040222 103	SRS878726	SAMN03436462	Golden retriever	B-cell
CCB040497 201	SRS878883	SAMN03436551	Golden retriever	Normal
CCB040497 102	SRS878845	SAMN03436513	Golden retriever	B-cell
CCB050193 200	SRS878871	SAMN03436539	Golden retriever	Normal
CCB050193 100	SRS878925	SAMN03436592	Golden retriever	B-cell
CCB050206 200	SRS879189	SAMN03436830	Golden retriever	Normal
CCB050206 102	SRS878585	SAMN03436269	Golden retriever	B-cell
CCB060047 201	SRS878869	SAMN03436537	Golden retriever	Normal
CCB060047 100	SRS878862	SAMN03436530	Golden retriever	B-cell
CCB060052 200	SRS878991	SAMN03436649	Golden retriever	Normal
CCB060052 104	SRS878831	SAMN03436504	Golden retriever	B-cell
CCB060153 200	SRS879128	SAMN03436787	Golden retriever	Normal
CCB060153 104	SRS878747	SAMN03436483	Golden retriever	B-cell
CCB070012 200	SRS879205	SAMN03436850	Golden retriever	Normal
CCB070012 104	SRS878879	SAMN03436547	Golden retriever	B-cell
CCB070081 201	SRS879123	SAMN03436782	Golden retriever	Normal
CCB070081 103	SRS878921	SAMN03436588	Golden retriever	B-cell
CCB070115 200	SRS879106	SAMN03436731	Golden retriever	Normal
CCB070115 100	SRS879102	SAMN03436727	Golden retriever	B-cell
CCB070230 201	SRS879042	SAMN03436699	Golden retriever	Normal
CCB070230 102	SRS879045	SAMN03436702	Golden retriever	B-cell
CCB070243 0200	SRS878980	SAMN03436638	Golden retriever	Normal
CCB070243 0103	SRS878717	SAMN03436453	Golden retriever	B-cell
CCB070252 201	SRS878743	SAMN03436479	Golden retriever	Normal
CCB070252 101	SRS878854	SAMN03436522	Golden retriever	B-cell
CCB070280 200	SRS879137	SAMN03436805	Golden retriever	Normal
CCB070280 102	SRS878990	SAMN03436648	Golden retriever	B-cell
MB2A11_Normal	SRS700802	SAMN03067895	Golden retriever	Normal
MB2A11_Tumor	SRS700801	SAMN03067894	Golden retriever	B-cell
MB2C13_Normal	SRS700797	SAMN03067893	Golden retriever	Normal
MB2C13_Tumor	SRS700803	SAMN03067896	Golden retriever	B-cell
MB140_Normal	SRS700794	SAMN03067891	Golden retriever	Normal
MB140_Tumor	SRS700780	SAMN03067879	Golden retriever	B-cell
BitRic_Normal	SRS1044184	SAMN04009639	Golden retriever	Normal
BitRic_Tumor	SRS1044176	SAMN04009635	Golden retriever	B-cell
BelBer_Normal	SRS1040992	SAMN04002390	Golden retriever	Normal
BelBer_Tumor	SRS1040987	SAMN04002385	Golden retriever	B-cell
BenGal_Normal	SRS1041025	SAMN04002435	Golden retriever	Normal
BenGal_Tumor	SRS1040999	SAMN04002423	Golden retriever	B-cell
BenTuc_Normal	SRS1044215	SAMN04009644	Golden retriever	Normal

BenTuc_Tumor	SRS1044177	SAMN04009636	Golden retriever	B-cell
CasLab_Normal	SRS1041010	SAMN04002429	Golden retriever	Normal
CasLab_Tumor	SRS1040997	SAMN04002421	Golden retriever	B-cell
ChiMun_Normal	SRS1044186	SAMN04009641	Golden retriever	Normal
ChiMun_Tumor	SRS1044178	SAMN04009637	Golden retriever	B-cell
JakRan_Normal	SRS1040998	SAMN04002422	Golden retriever	Normal
JakRan_Tumor	SRS1040995	SAMN04002419	Golden retriever	B-cell
MadGus_Normal	SRS1041001	SAMN04002425	Golden retriever	Normal
MadGus_Tumor	SRS1040996	SAMN04002420	Golden retriever	B-cell
MBGRD14_Normal	SRS1041019	SAMN04002431	Golden retriever	Normal
MBGRD14_Tumor	SRS1041003	SAMN04002426	Golden retriever	B-cell
MBGRF16_Normal	SRS1041029	SAMN04002437	Golden retriever	Normal
MBGRF16_Tumor	SRS1040990	SAMN04002388	Golden retriever	B-cell
N149867_Normal	SRS1040989	SAMN04002387	Golden retriever	Normal
N149867_Tumor	SRS1041015	SAMN04002430	Golden retriever	B-cell
N150309_Normal	SRS1041020	SAMN04002432	Golden retriever	Normal
N150309_Tumor	SRS1041006	SAMN04002428	Golden retriever	B-cell
PabWil_Normal	SRS1041021	SAMN04002433	Golden retriever	Normal
PabWil_Tumor	SRS1041023	SAMN04002434	Golden retriever	B-cell
RaiEva_Normal	SRS1040994	SAMN04002392	Golden retriever	Normal
RaiEva_Tumor	SRS1041005	SAMN04002427	Golden retriever	B-cell
RenJoh_Normal	SRS1044187	SAMN04009642	Golden retriever	Normal
RenJoh_Tumor	SRS1044183	SAMN04009638	Golden retriever	B-cell
StaMur_Normal	SRS1044188	SAMN04009643	Golden retriever	Normal
StaMur_Tumor	SRS1044185	SAMN04009640	Golden retriever	B-cell
CCB010233 201	SRS878996	SAMN03436654	Golden retriever	Normal
CCB010233 102	SRS878971	SAMN03436629	Golden retriever	T-cell
CCB010243 200	SRS879105	SAMN03436730	Golden retriever	Normal
CCB010243 104	SRS879038	SAMN03436695	Golden retriever	T-cell
CCB010253 201	SRS878884	SAMN03436552	Golden retriever	Normal
CCB010253 101	SRS878888	SAMN03436556	Golden retriever	T-cell
CCB010289 201	SRS878895	SAMN03436562	Golden retriever	Normal
CCB010289 104	SRS879002	SAMN03436660	Golden retriever	T-cell
CCB030003 200	SRS879441	SAMN03436934	Golden retriever	Normal
CCB030003 102	SRS878960	SAMN03436618	Golden retriever	T-cell
CCB030016 201	SRS879019	SAMN03436677	Golden retriever	Normal
CCB030016 104	SRS878751	SAMN03436487	Golden retriever	T-cell
CCB030026 201	SRS879210	SAMN03436854	Golden retriever	Normal
CCB030026 100	SRS879018	SAMN03436676	Golden retriever	T-cell
CCB030059 201	SRS879433	SAMN03436926	Golden retriever	Normal
CCB030059 100	SRS879400	SAMN03436893	Golden retriever	T-cell
CCB030156 200	SRS879197	SAMN03436838	Golden retriever	Normal
CCB030156 100	SRS878848	SAMN03436516	Golden retriever	T-cell
CCB030258 200	SRS878864	SAMN03436532	Golden retriever	Normal
CCB030258 101	SRS878665	SAMN03436285	Golden retriever	T-cell
CCB030259 200	SRS879190	SAMN03436831	Golden retriever	Normal

CCB030259 104	SRS878700	SAMN03436296	Golden retriever	T-cell
CCB030265 201	SRS879025	SAMN03436683	Golden retriever	Normal
CCB030265 102	SRS878852	SAMN03436520	Golden retriever	T-cell
CCB030271 201	SRS878954	SAMN03436612	Golden retriever	Normal
CCB030271 102	SRS878977	SAMN03436635	Golden retriever	T-cell
CCB030300 201	SRS879212	SAMN03436856	Golden retriever	Normal
CCB030300 102	SRS878905	SAMN03436572	Golden retriever	T-cell
CCB030317 200	SRS879183	SAMN03436824	Golden retriever	Normal
CCB030317 100	SRS878773	SAMN03436498	Golden retriever	T-cell
CCB040292 201	SRS879396	SAMN03436889	Golden retriever	Normal
CCB040292 104	SRS878976	SAMN03436634	Golden retriever	T-cell
CCB040425 201	SRS878995	SAMN03436653	Golden retriever	Normal
CCB040425 102	SRS878775	SAMN03436500	Golden retriever	T-cell
CCB060008 200	SRS879071	SAMN03436714	Golden retriever	Normal
CCB060008 100	SRS878894	SAMN03436561	Golden retriever	T-cell
CCB060151	SRS879109	SAMN03436734	Golden retriever	Normal
CCB060151 104	SRS878872	SAMN03436540	Golden retriever	T-cell
CCB070080 0201	SRS878945	SAMN03436605	Golden retriever	Normal
CCB070080 0101	SRS878697	SAMN03436293	Golden retriever	T-cell
CCB070139 0201	SRS878889	SAMN03436557	Golden retriever	Normal
CCB070139 0100	SRS879187	SAMN03436828	Golden retriever	T-cell
CCB070162 0200	SRS879108	SAMN03436733	Golden retriever	Normal
CCB070162 0101	SRS878481	SAMN03436221	Golden retriever	T-cell
CCB070292 0201	SRS878909	SAMN03436576	Golden retriever	Normal
CCB070292 0101	SRS878958	SAMN03436616	Golden retriever	T-cell
NEWMET_Normal	SRS700779	SAMN03067878	Golden retriever	Normal
NEWMET_Tumor	SRS700781	SAMN03067880	Golden retriever	T-cell
SHAKIT_Normal	SRS700773	SAMN03067876	Golden retriever	Normal
SHAKIT_Tumor	SRS700782	SAMN03067881	Golden retriever	T-cell
