# Supplementary material for: Exome sequencing of lymphomas from three dog breeds reveals somatic mutation patterns reflecting genetic background
Source: Genome Res. 2015 Nov;25(11):1634–45. doi: 10.1101/gr.194449.115 (PMC4617960; doi:10.1101/gr.194449.115)
Supplement: Supplemental Material [file supp_gr.194449.115_Supp_Table2.pdf]

Supplementary Table 2. All significantly mutated genes. Multiple mutations in one gene in one individual are counted as one. CT, convolution test.

Cocker spaniel, B-cell

Gene	geneID	P-value CT	FDR CT
ENSCAFG00000030674	*FAM90A1*	3.47E-09	8.51E-05
ENSCAFG00000014251	*DDX3X*	1.72E-08	2.10E-04
ENSCAFG00000018075	*TRAF3*	6.44E-08	5.26E-04
ENSCAFG00000006496	*MITF*	1.70E-07	1.04E-03
ENSCAFG00000001396	*PSMA1*	4.11E-07	2.02E-03
ENSCAFG00000001086	*MYC*	1.46E-06	5.98E-03
ENSCAFG00000001754	*GRIFIN*	6.07E-06	2.13E-02
ENSCAFG00000016403	*POT1*	8.15E-06	2.22E-02

Golden retriever, B-cell

Gene	geneID	P-value CT	FDR CT
ENSCAFG00000008141	*FBXW7*	0.00E+00	0.00E+00
ENSCAFG00000001754	*POT1*	1.10E-22	1.34E-18
ENSCAFG00000018075	*TRAF3*	1.56E-19	1.28E-15
ENSCAFG00000030674	*FAM90A1*	2.36E-18	1.45E-14
ENSCAFG00000031638	*ENSCAFG000*	5.30E-18	2.60E-14
ENSCAFG00000016714	*TP53*	2.25E-17	9.18E-14
ENSCAFG00000030839	*PNRC1*	1.07E-12	3.76E-09
ENSCAFG00000000578	*FKBP3*	1.26E-09	3.32E-06
ENSCAFG00000025100	*TBC1D26*	1.13E-09	3.32E-06
ENSCAFG00000017753	*RPL23A*	1.35E-09	3.32E-06
ENSCAFG00000025123	*FAM90A1*	6.43E-09	1.43E-05
ENSCAFG00000006180	*SOCS2*	7.75E-09	1.58E-05
ENSCAFG00000001396	*PSMA1*	1.21E-08	2.28E-05
ENSCAFG00000014251	*DDX3X*	1.99E-08	3.48E-05
ENSCAFG00000013392	*SETD2*	3.33E-08	5.44E-05
ENSCAFG00000013774	*MAP3K14*	1.50E-07	2.17E-04
ENSCAFG00000005151	*BRK1*	3.73E-07	5.08E-04
ENSCAFG00000029820	*ENSCAFG000*	4.89E-07	6.31E-04
ENSCAFG00000017032	*ENSCAFG000*	7.47E-07	9.16E-04
ENSCAFG00000030482	*ENSCAFG000*	8.30E-07	9.69E-04
ENSCAFG00000030950	*ENSCAFG000*	1.46E-06	1.63E-03
ENSCAFG00000013453	*KLRK1*	1.88E-06	2.00E-03
ENSCAFG00000010684	*LINS*	1.99E-06	2.04E-03
ENSCAFG00000014042	*KLRK1*	2.27E-06	2.22E-03
ENSCAFG00000003796	*MAPRE1*	2.39E-06	2.25E-03
ENSCAFG00000014958	*RPL22*	3.15E-06	2.86E-03

ENSCAFG00000009912	*ERG*	3.79E-06	3.31E-03
ENSCAFG00000028928	*OR5AC1*	4.74E-06	4.01E-03
ENSCAFG00000017316	*ENSCAFG000*	5.63E-06	4.61E-03
ENSCAFG00000013551	*ATP5H*	6.02E-06	4.76E-03
ENSCAFG00000029241	*ENSCAFG000*	6.53E-06	4.85E-03
ENSCAFG00000029904	*HBA1*	6.39E-06	4.85E-03
ENSCAFG00000016289	*CTB-167G5.5*	7.40E-06	5.33E-03
ENSCAFG00000011598	*KPNA2*	1.03E-05	7.20E-03
ENSCAFG00000019962	*TAF1C*	1.15E-05	7.80E-03
ENSCAFG00000013060	*PDK1*	1.21E-05	7.82E-03
ENSCAFG00000016528	*NTRK1*	1.21E-05	7.82E-03
ENSCAFG00000032009	*ENSCAFG000*	1.35E-05	8.51E-03
ENSCAFG00000000595	*ENSCAFG000*	1.46E-05	8.92E-03
ENSCAFG00000016251	*EMR2*	1.57E-05	9.39E-03
ENSCAFG00000030976	*ENSCAFG000*	1.83E-05	1.07E-02
ENSCAFG00000010012	*ST13*	1.90E-05	1.09E-02
ENSCAFG00000024522	*ENSCAFG000*	1.99E-05	1.11E-02
ENSCAFG00000032455	*ENSCAFG000*	2.07E-05	1.13E-02
ENSCAFG00000009973	*EEF1A2*	2.16E-05	1.15E-02
ENSCAFG00000006036	*HNRNPA1P1(*	2.33E-05	1.21E-02
ENSCAFG00000025327	*SOX17*	2.40E-05	1.22E-02
ENSCAFG00000001336	*ISCA1*	2.51E-05	1.26E-02
ENSCAFG00000030845	*HIST2H3PS2*	2.62E-05	1.29E-02
ENSCAFG00000014107	*RPL23A*	3.23E-05	1.52E-02
ENSCAFG00000018032	*C16orf88*	3.91E-05	1.81E-02
ENSCAFG00000029913	*ENSCAFG000*	6.40E-05	2.85E-02
ENSCAFG00000019424	*PKD1*	6.68E-05	2.92E-02
ENSCAFG00000006873	*ZDHHC2*	7.21E-05	3.05E-02
ENSCAFG00000004640	*EEF1A1*	7.21E-05	3.05E-02
ENSCAFG00000006663	*intron of SNX*	7.78E-05	3.23E-02
ENSCAFG00000031340	*ENSCAFG000*	9.09E-05	3.60E-02
ENSCAFG00000005959	*CWC15*	8.99E-05	3.60E-02
ENSCAFG00000020326	*DUS2L*	8.90E-05	3.60E-02
ENSCAFG00000000039	*DOK6*	9.63E-05	3.75E-02
ENSCAFG00000005185	*XRN2*	9.91E-05	3.80E-02
ENSCAFG00000024545	*PDIA6*	1.09E-04	3.90E-02
ENSCAFG00000014786	*PHC3*	1.09E-04	3.90E-02
ENSCAFG00000031200	*ENSCAFG000*	1.06E-04	3.90E-02
ENSCAFG00000008450	*WDR17*	1.10E-04	3.90E-02
ENSCAFG00000028889	*USP17L2*	1.05E-04	3.90E-02
ENSCAFG00000003493	*ENSCAFG000*	1.21E-04	4.22E-02
ENSCAFG00000004229	*GLUD2*	1.26E-04	4.28E-02

ENSCAFG00000008814	*ENSCAFG000*	1.25E-04	4.28E-02
ENSCAFG00000006166	*MDH2*	1.51E-04	4.99E-02
ENSCAFG00000031827	*ZNF486*	1.50E-04	4.99E-02

Boxer, T-cell

Gene	geneID	P-value CT	FDR CT
ENSCAFG00000015670	*PTEN*	2.52E-11	6.19E-07
ENSCAFG00000005839	*SATB1*	3.61E-10	4.42E-06
ENSCAFG00000017298	*MAP2K1*	2.28E-06	1.86E-02
ENSCAFG00000006937	*EEF1A1*	6.40E-06	3.26E-02
ENSCAFG00000006714	*NLRP14*	6.64E-06	3.26E-02
ENSCAFG00000024216	*KCND2*	1.22E-05	4.99E-02

Golden retriever, T-cell

Gene	geneID	P-value CT	FDR CT
ENSCAFG00000001396	*PSMA1*	4.86E-13	1.19E-08
ENSCAFG00000024399	*COX8A*	1.51E-11	1.85E-07
ENSCAFG00000006440	*LTA4H*	1.95E-09	1.19E-05
ENSCAFG00000025100	*TBC1D26*	1.57E-09	1.19E-05
ENSCAFG00000000584	*ZNF706*	1.77E-08	8.69E-05
ENSCAFG00000013551	*ATP5H*	7.49E-08	3.06E-04
ENSCAFG00000031638	*ENSCAFG000*	1.45E-07	5.09E-04
ENSCAFG00000014463	*PTPN6*	3.23E-07	9.91E-04
ENSCAFG00000002501	*NLRP5*	3.68E-07	1.00E-03
ENSCAFG00000004229	*GLUD2*	6.04E-07	1.48E-03
ENSCAFG00000013232	*RPL11*	6.92E-07	1.54E-03
ENSCAFG00000005839	*SATB1*	3.44E-06	7.04E-03
ENSCAFG00000017753	*RPL23A*	4.08E-06	7.70E-03
ENSCAFG00000011017	*KRTAP10-6*	5.13E-06	8.98E-03
ENSCAFG00000019029	*MAGEC2*	6.38E-06	1.04E-02
ENSCAFG00000002232	*HNRNPA1L2*	1.05E-05	1.61E-02
ENSCAFG00000029913	*ENSCAFG000*	1.73E-05	2.23E-02
ENSCAFG00000011895	*PTAFR*	1.59E-05	2.23E-02
ENSCAFG00000008141	*FBXW7*	2.19E-05	2.68E-02
ENSCAFG00000015252	*NME7*	2.97E-05	3.47E-02

All B-cell (cocker spaniel + golden retriever)

Gene	geneID	P-value CT	FDR CT
ENSCAFG00000001754	*POT1*	0.00E+00	0.00E+00
ENSCAFG00000008141	*FBXW7*	0.00E+00	0.00E+00
ENSCAFG00000018075	*TRAF3*	0.00E+00	0.00E+00
ENSCAFG00000030674	*FAM90A1*	0.00E+00	0.00E+00

ENSCAFG00000016714	*TP53*	1.25E-22	6.11E-19
ENSCAFG00000031638	*ENSCAFG000*	2.62E-20	1.07E-16
ENSCAFG00000014251	*DDX3X*	7.44E-15	2.60E-11
ENSCAFG00000001396	*PSMA1*	4.69E-14	1.44E-10
ENSCAFG00000017753	*RPL23A*	2.72E-12	7.03E-09
ENSCAFG00000030839	*PNRC1*	2.87E-12	7.03E-09
ENSCAFG00000013392	*SETD2*	2.42E-11	5.40E-08
ENSCAFG00000013774	*MAP3K14*	1.10E-10	2.24E-07
ENSCAFG00000000578	*FKBP3*	3.37E-09	5.90E-06
ENSCAFG00000025100	*TBC1D26*	3.17E-09	5.90E-06
ENSCAFG00000006180	*SOCS2*	1.17E-08	1.91E-05
ENSCAFG00000010684	*LINS*	1.39E-08	2.02E-05
ENSCAFG00000028928	*OR5AC1*	1.40E-08	2.02E-05
ENSCAFG00000025123	*FAM90A1*	1.66E-08	2.26E-05
ENSCAFG00000009912	*ERG*	3.25E-08	4.19E-05
ENSCAFG00000006036	*HNRNPA1P1(*	1.45E-07	1.77E-04
ENSCAFG00000009973	*EEF1A2*	1.70E-07	1.98E-04
ENSCAFG00000030482	*ENSCAFG000*	7.76E-07	7.81E-04
ENSCAFG00000028889	*USP17L2*	7.34E-07	7.81E-04
ENSCAFG00000029820	*ENSCAFG000*	7.96E-07	7.81E-04
ENSCAFG00000005151	*BRK1*	1.02E-06	9.63E-04
ENSCAFG00000017032	*ENSCAFG000*	1.33E-06	1.21E-03
ENSCAFG00000030950	*ENSCAFG000*	2.11E-06	1.85E-03
ENSCAFG00000013453	*KLRK1*	2.27E-06	1.92E-03
ENSCAFG00000003297	*LRRN3*	2.37E-06	1.94E-03
ENSCAFG00000014042	*KLRK1*	3.71E-06	2.93E-03
ENSCAFG00000019424	*PKD1*	5.56E-06	4.13E-03
ENSCAFG00000003796	*MAPRE1*	5.41E-06	4.13E-03
ENSCAFG00000000608	*ARID1B*	6.57E-06	4.74E-03
ENSCAFG00000015326	*SLC38A5*	7.84E-06	5.27E-03
ENSCAFG00000029241	*ENSCAFG000*	8.38E-06	5.27E-03
ENSCAFG00000014958	*RPL22*	8.33E-06	5.27E-03
ENSCAFG00000013551	*ATP5H*	7.79E-06	5.27E-03
ENSCAFG00000000749	*ENSCAFG000*	8.30E-06	5.27E-03
ENSCAFG00000029054	*ENSCAFG000*	1.15E-05	6.88E-03
ENSCAFG00000017316	*ENSCAFG000*	1.15E-05	6.88E-03
ENSCAFG00000016289	*CTB-167G5.5*	1.27E-05	7.30E-03
ENSCAFG00000029904	*HBA1*	1.28E-05	7.30E-03
ENSCAFG00000019962	*TAF1C*	1.70E-05	9.19E-03
ENSCAFG00000011598	*KPNA2*	1.65E-05	9.19E-03
ENSCAFG00000016528	*NTRK1*	1.72E-05	9.19E-03
ENSCAFG00000000595	*ENSCAFG000*	1.99E-05	1.04E-02

ENSCAFG00000032009	*ENSCAFG000*	2.20E-05	1.12E-02
ENSCAFG00000010012	*ST13*	2.29E-05	1.13E-02
ENSCAFG00000025327	*SOX17*	2.31E-05	1.13E-02
ENSCAFG00000006496	*MITF*	2.39E-05	1.15E-02
ENSCAFG00000032455	*ENSCAFG000*	2.51E-05	1.19E-02
ENSCAFG00000024522	*ENSCAFG000*	2.64E-05	1.22E-02
ENSCAFG00000016251	*EMR2*	2.89E-05	1.31E-02
ENSCAFG00000001336	*ISCA1*	3.09E-05	1.38E-02
ENSCAFG00000017103	*TAF1L*	3.81E-05	1.67E-02
ENSCAFG00000030845	*HIST2H3PS2*	3.95E-05	1.70E-02
ENSCAFG00000029913	*ENSCAFG000*	4.32E-05	1.83E-02
ENSCAFG00000014107	*ENSCAFG000*	4.67E-05	1.94E-02
ENSCAFG00000013060	*PDK1*	6.39E-05	2.53E-02
ENSCAFG00000018032	*C16orf88*	7.02E-05	2.73E-02
ENSCAFG00000017134	*OGT*	9.24E-05	3.49E-02
ENSCAFG00000031340	*ENSCAFG000*	9.15E-05	3.49E-02
ENSCAFG00000001086	*MYC*	9.87E-05	3.67E-02
ENSCAFG00000031200	*ENSCAFG000*	1.07E-04	3.91E-02
ENSCAFG00000014786	*PHC3*	1.22E-04	4.38E-02
ENSCAFG00000005959	*CWC15*	1.28E-04	4.53E-02
ENSCAFG00000006873	*ZDHHC2*	1.32E-04	4.55E-02
ENSCAFG00000013599	*HRG*	1.31E-04	4.55E-02
ENSCAFG00000004640	*ENSCAFG000*	1.38E-04	4.62E-02
ENSCAFG00000016403	*GRIFIN*	1.37E-04	4.62E-02
ENSCAFG00000003493	*ENSCAFG000*	1.42E-04	4.69E-02
ENSCAFG00000024545	*ENSCAFG000*	1.55E-04	4.88E-02
ENSCAFG00000000039	*DOK6*	1.56E-04	4.88E-02
ENSCAFG00000005185	*XRN2*	1.59E-04	4.88E-02
ENSCAFG00000031054	*ENSCAFG000*	1.58E-04	4.88E-02
ENSCAFG00000006663	*ENSCAFG000*	1.59E-04	4.88E-02
ENSCAFG00000020326	*DUS2L*	1.58E-04	4.88E-02

All T-cell (boxer + golden retriever)

Gene	geneID	P-value CT	FDR CT
ENSCAFG00000005839	*SATB1*	2.40E-15	5.88E-11
ENSCAFG00000025100	*TBC1D26*	2.99E-14	3.67E-10
ENSCAFG00000001396	*PSMA1*	1.38E-11	1.12E-07
ENSCAFG00000024399	*COX8A*	7.06E-11	4.33E-07
ENSCAFG00000031638	*ENSCAFG000*	3.27E-09	1.55E-05
ENSCAFG00000015670	*PTEN*	3.79E-09	1.55E-05
ENSCAFG00000006440	*LTA4H*	2.73E-08	9.57E-05
ENSCAFG00000000584	*ZNF706*	6.80E-08	2.08E-04

ENSCAFG00000013551	*ATP5H*	2.81E-07	7.67E-04
ENSCAFG00000029967	*RPLP2*	9.15E-07	2.24E-03
ENSCAFG00000005019	*GATA3*	1.33E-06	2.98E-03
ENSCAFG00000002501	*NLRP5*	2.14E-06	4.37E-03
ENSCAFG00000014463	*PTPN6*	2.44E-06	4.59E-03
ENSCAFG00000004229	*GLUD2*	3.81E-06	5.49E-03
ENSCAFG00000008369	*PBK*	3.49E-06	5.49E-03
ENSCAFG00000016310	*IKZF3*	3.62E-06	5.49E-03
ENSCAFG00000013232	*RPL11*	4.03E-06	5.49E-03
ENSCAFG00000017753	*RPL23A*	7.22E-06	9.32E-03
ENSCAFG00000030674	*FAM90A1*	8.35E-06	1.02E-02
ENSCAFG00000017298	*MAP2K1*	1.39E-05	1.63E-02
ENSCAFG00000029913	*ENSCAFG000*	1.71E-05	1.90E-02
ENSCAFG00000019029	*MAGEC2*	2.22E-05	2.26E-02
ENSCAFG00000002232	*HNRNPA1L2*	2.14E-05	2.26E-02
ENSCAFG00000011017	*KRTAP10-6*	2.33E-05	2.29E-02
