# Supplementary material for: Exome sequencing of lymphomas from three dog breeds reveals somatic mutation patterns reflecting genetic background
Source: Genome Res. 2015 Nov;25(11):1634–45. doi: 10.1101/gr.194449.115 (PMC4617960; doi:10.1101/gr.194449.115)
Supplement: Supplemental Material [file supp_gr.194449.115_Supp_Table3.pdf]

Supplementary Table 3a. Protein identity between top significantly mutated canine genes and their human counterparts.

Canine Ensembl gene ID	Human Ensembl gene ID	% protein identity with respect to canine gene	% protein identity with respect to human gene	Human common name	Comment
ENSCAFG00000000578	*ENSG00000100442*	92	92	*FKBP3*	
ENSCAFG00000000584	*ENSG00000120963*	100	100	*ZNF706*	
ENSCAFG00000001086	*ENSG00000136997*	94	91	*MYC*	
ENSCAFG00000001396	*ENSG00000129084*	96	94	*PSMA1*	
ENSCAFG00000001754	*ENSG00000128513*	72	89	*GRIFIN*	
ENSCAFG00000002501	*ENSG00000171487*	63	62	*NLRP5*	
ENSCAFG00000004229	*ENSG00000182890*	92	83	*GLUD2*	
ENSCAFG00000005839	*ENSG00000182568*	87	76	*SATB1*	
ENSCAFG00000006180	*ENSG00000120833*	93	94	*SOCS2*	
ENSCAFG00000006440	*ENSG00000111144*	83	95	*LTA4H*	
ENSCAFG00000006496	*ENSG00000187098*	94	86	*MITF*	
ENSCAFG00000006714	*ENSG00000158077*	77	77	*NLRP14*	
ENSCAFG00000006937	*ENSG00000196205*	85	85	*EEF1A1*	human gene is a pseudogene
ENSCAFG00000008141	*ENSG00000109670*	98	99	*FBXW7*	
ENSCAFG00000011017	*ENSG00000188155*	65	50	*KRTAP10-6*	exact human ortholog unclear
ENSCAFG00000013232	*ENSG00000142676*	100	100	*RPL11*	
ENSCAFG00000013392	*ENSG00000181555*	92	92	*SETD2*	
ENSCAFG00000013551	*ENSG00000167863*	89	83	*ATP5H*	
ENSCAFG00000014251	*ENSG00000215301*	94	100	*DDX3X*	
ENSCAFG00000014463	*ENSG00000111679*	91	87	*PTPN6*	
ENSCAFG00000015670	*ENSG00000171862*	99	94	*PTEN*	
ENSCAFG00000016403	*ENSG00000275572*	77	77	*POT1*	

ENSCAFG00000016714	*ENSG00000141510*	82	80	*TP53*	
ENSCAFG00000017298	*ENSG00000169032*	99	99	*MAP2K1*	
ENSCAFG00000017753	*ENSG00000234851*	67	65	*RPL23A*	human gene is a pseudogene
ENSCAFG00000018075	*ENSG00000131323*	96	96	*TRAF3*	
ENSCAFG00000019029	*ENSG00000046774*	36	35	*MAGEC2*	may be pseudogene; exact human ortholog unclear
ENSCAFG00000024216	*ENSG00000184408*	98	58	*KCND2*	
ENSCAFG00000024399	*ENSG00000176340*	65	65	*COX8A*	
ENSCAFG00000025100	*ENSG00000214946*	19	28	*TBC1D26*	
ENSCAFG00000025123	*ENSG00000171847*	34	33	*FAM90A1*	*ENSCAFG00000025123* and *ENSCAFG00000030674* are both listed as *ENSG00000171847* orthologs in Ensembl. The mutations in those genes cluster, suggesting they are not pseudogenes accumulating random mutations.
ENSCAFG00000030674	*ENSG00000171847*	36	35	*FAM90A1*	
ENSCAFG00000030839	*ENSG00000146278*	90	90	*PNRC1*	
ENSCAFG00000031638	antisense against part of *ENSG00000167608*			antisense in *TMC4*	intron 1, exon 2, intron 2

Supplementary Table 3b. FBXW7 alignment. Position R465 (human) and R470 (dog) highlighted in blue.

```
FBXW7 hum   1     MNOELLSVGSKRRRTGGSLRGNPSSSOVDEEOMNRVVEEEOOOO-----LROOEEEHTAR  55
                  MNQELLSVGSKRRRTGGSLRGNPSSSQ DEEQMNRVVEEEQQQQ     LRQQEEEHTAR
FBXW7_can   1     MNQELLSVGSKRRRTGGSLRGNPSSSQADEEQMNRVVEEEQQQQQQQQQLRQQEEEHTAR  60

FBXW7 hum   56    NGEVVGVEPRPGGONDSOOGOLEENNNRFISVDEDSSGNOEEOEEDEEHAGEODEEDEEE  115
                  NGEVVG EPRPG QNDSQQG LEENNNRFISVDEDSSGNQEEQEEDEEHAGEQDEEDEEE
FBXW7_can   61    NGEVVGAEPRPGDQNDSQQGHLEENNNRFISVDEDSSGNQEEQEEDEEHAGEQDEEDEEE  120

FBXW7 hum   116   EEMDOESDDFDOSDDSSREDEHTHTNSVTNSSSIVDLPVHOLSSPFYTKTTKMKRKLDHG  175
                  EEMDQESDDFDQSDDSSRED+HTH+NSVTNS+SIVDLP+HQLSSPFYTKTTKMKRKLDHG
FBXW7_can   121   EEMDQESDDFDQSDDSSREDDHTHSNSVTNSTSIVDLPIHQLSSPFYTKTTKMKRKLDHG  180

FBXW7 hum   176   SEVRSFSLGKKPCKVSEYTSTTGLVPCSATPTTFGDLRAANGOGOORRRITSVOPPTGLO  235
                  SEVRSFSLGKKPCKVSEYTSTTGLVPCSATPTTFGDLRAANGQGQQRRRITSVQPPTGLQ
FBXW7_can   181   SEVRSFSLGKKPCKVSEYTSTTGLVPCSATPTTFGDLRAANGQGQQRRRITSVQPPTGLQ  240

FBXW7 hum   236   EWLKMFOSWSGPEKLLALDELIDSCEPTOVKHMMOVIEPOFORDFISLLPKELALYVLSF  295
                  EWLKMFQSWSGPEKLLALDELIDSCEPTQVKHMMQVIEPQFQRDFISLLPKELALYVLSF
FBXW7_can   241   EWLKMFQSWSGPEKLLALDELIDSCEPTQVKHMMQVIEPQFQRDFISLLPKELALYVLSF  300

FBXW7 hum   296   LEPKDLLOAAOTCRYWRILAEDNLLWREKCKEEGIDEPLHIKRRKVIKPGFIHSPWKSAY  355
                  LEPKDLLQAAQTCRYWRILAEDNLLWREKCKEEGIDEPLHIKRRKVIKPGFIHSPWKSAY
FBXW7_can   301   LEPKDLLQAAQTCRYWRILAEDNLLWREKCKEEGIDEPLHIKRRKVIKPGFIHSPWKSAY  360

FBXW7 hum   356   IROHRIDTNWRRGELKSPKVLKGHDDHVITCLOFCGNRIVSGSDDNTLKVWSAVTGKCLR  415
                  IRQHRIDTNWRRGELKSPKVLKGHDDHVITCLQFCGNRIVSGSDDNTLKVWSAVTGKCLR
FBXW7_can   361   IRQHRIDTNWRRGELKSPKVLKGHDDHVITCLQFCGNRIVSGSDDNTLKVWSAVTGKCLR  420

FBXW7 hum   416   TLVGHTGGVWSSOMRDNIIISGSTDRTLKVWNAETGECIHTLYGHTSTVRCMHLHEKRVV  475
                  TLVGHTGGVWSSQMRDNIIISGSTDRTLKVWNAETGECIHTLYGHTSTVRCMHLHEKRVV
FBXW7_can   421   TLVGHTGGVWSSQMRDNIIISGSTDRTLKVWNAETGECIHTLYGHTSTVRCMHLHEKRVV  480

FBXW7 hum   476   SGSRDATLRVWDIETGOCLHVLMGHVAAVRCVOYDGRRVVSGAYDFMVKVWDPETETCLH  535
```

```
                   SGSRDATLRVWDIETGQCLHVLMGHVAAVRCVQYDGRRVVSGAYDFMVKVWDPETETCLH
FBXW7_can   481    SGSRDATLRVWDIETGQCLHVLMGHVAAVRCVQYDGRRVVSGAYDFMVKVWDPETETCLH   540

FBXW7_hum   536    TLQGHTNRVYSLQFDGIHVVSGSLDTSIRVWDVETGNCIHTLTGHQSLTSGMELKDNILV   595
                   TLQGHTNRVYSLQFDGIHVVSGSLDTSIRVWDVETGNCIHTLTGHQSLTSGMELKDNILV
FBXW7_can   541    TLQGHTNRVYSLQFDGIHVVSGSLDTSIRVWDVETGNCIHTLTGHQSLTSGMELKDNILV   600

FBXW7_hum   596    SGNADSTVKIWDIKTGQCLQTLQGPNKHQSAVTCLQFNKNFVITSSDDGTVKLWDLKTGE   655
                   SGNADSTVKIWDIKTGQCLQTLQGPNKHQSAVTCLQFNKNFVITSSDDGTVKLWDLKTGE
FBXW7_can   601    SGNADSTVKIWDIKTGQCLQTLQGPNKHQSAVTCLQFNKNFVITSSDDGTVKLWDLKTGE   660

FBXW7_hum   656    FIRNLVTLESGGSGGVVWRIRASNTKLVCAVGSRNGTEETKLLVLDFDVDMK   707
                   FIRNLVTLESGGSGGVVWRIRASNTKLVCAVGSRNGTEETKLLVLDFDVDMK
FBXW7_can   661    FIRNLVTLESGGSGGVVWRIRASNTKLVCAVGSRNGTEETKLLVLDFDVDMK   712
```
