# Supplementary material for: Exome sequencing of lymphomas from three dog breeds reveals somatic mutation patterns reflecting genetic background
Source: Genome Res. 2015 Nov;25(11):1634–45. doi: 10.1101/gr.194449.115 (PMC4617960; doi:10.1101/gr.194449.115)
Supplement: Supplemental Material [file supp_gr.194449.115_Supp_Table4.pdf]

Supplementary Table 4. IPA-suggested canonical pathways ($p<0.05$) for significantly mutated genes from B-cell lymphomas from cocker spaniel, golden retriever, and both breeds combined

Cocker spaniel

Ingenuity Canonical Pathways	p-value	Ratio	Genes
Small Cell Lung Cancer Signaling	2.57E-04	2.82E-02	*MYC,TRAF3*
Telomerase Signaling	5.01E-04	2.02E-02	*MYC,POT1*
Role of Macrophages, Fibroblasts and Endothelial Cells in Rheumatoid Arthritis	4.37E-03	6.71E-03	*MYC,TRAF3*
Telomere Extension by Telomerase	5.25E-03	6.67E-02	*POT1*
Polyamine Regulation in Colon Cancer	7.59E-03	4.55E-02	*MYC*
Estrogen-mediated S-phase Entry	8.32E-03	4.17E-02	*MYC*
TWEAK Signaling	1.17E-02	2.94E-02	*TRAF3*
April Mediated Signaling	1.32E-02	2.63E-02	*TRAF3*
Role of PKR in Interferon Induction and Antiviral Response	1.38E-02	2.50E-02	*TRAF3*
B Cell Activating Factor Signaling	1.38E-02	2.50E-02	*TRAF3*
Thyroid Cancer Signaling	1.38E-02	2.50E-02	*MYC*
Melanoma Signaling	1.45E-02	2.38E-02	*MITF*
Role of RIG1-like Receptors in Antiviral Innate Immunity	1.55E-02	2.22E-02	*TRAF3*
Endometrial Cancer Signaling	1.78E-02	1.92E-02	*MYC*
Lymphotoxin β Receptor Signaling	1.86E-02	1.85E-02	*TRAF3*
Thrombopoietin Signaling	1.91E-02	1.82E-02	*MYC*
ErbB2-ErbB3 Signaling	1.95E-02	1.75E-02	*MYC*
Myc Mediated Apoptosis Signaling	2.00E-02	1.72E-02	*MYC*
ERK5 Signaling	2.19E-02	1.59E-02	*MYC*
Activation of IRF by Cytosolic Pattern Recognition Receptors	2.19E-02	1.56E-02	*TRAF3*
Cell Cycle: G1/S Checkpoint Regulation	2.19E-02	1.56E-02	*MYC*
CD40 Signaling	2.24E-02	1.54E-02	*TRAF3*
Prolactin Signaling	2.51E-02	1.37E-02	*MYC*
STAT3 Pathway	2.51E-02	1.37E-02	*MYC*
Acute Myeloid Leukemia Signaling	2.63E-02	1.30E-02	*MYC*
PDGF Signaling	2.63E-02	1.30E-02	*MYC*
Melanocyte Development and Pigmentation Signaling	2.88E-02	1.19E-02	*MITF*
Bladder Cancer Signaling	3.02E-02	1.15E-02	*MYC*
Neuregulin Signaling	3.02E-02	1.14E-02	*MYC*
Altered T Cell and B Cell Signaling in Rheumatoid Arthritis	3.02E-02	1.14E-02	*TRAF3*
RANK Signaling in Osteoclasts	3.02E-02	1.14E-02	*MITF*

OX40 Signaling Pathway	3.09E-02	1.12E-02	*TRAF3*
Chronic Myeloid Leukemia Signaling	3.16E-02	1.08E-02	*MYC*
Mouse Embryonic Stem Cell Pluripotency	3.24E-02	1.05E-02	*MYC*
p38 MAPK Signaling	3.98E-02	8.55E-03	*MYC*
P2Y Purigenic Receptor Signaling Pathway	4.07E-02	8.40E-03	*MYC*
Aryl Hydrocarbon Receptor Signaling	4.79E-02	7.14E-03	*MYC*

Golden retriever

Ingenuity Canonical Pathways	p-value	Ratio	Genes
NGF Signaling	1.91E-03	2.80E-02	*TP53,MAP3K14,NTRK1*
TWEAK Signaling	2.75E-03	5.88E-02	*MAP3K14,TRAF3*
April Mediated Signaling	3.39E-03	5.26E-02	*MAP3K14,TRAF3*
Role of PKR in Interferon Induction and Antiviral Response	3.80E-03	5.00E-02	*TP53,TRAF3*
B Cell Activating Factor Signaling	3.80E-03	5.00E-02	*MAP3K14,TRAF3*
Thyroid Cancer Signaling	3.80E-03	5.00E-02	*TP53,NTRK1*
Glutamate Biosynthesis II	4.57E-03	5.00E-01	*GLUD2*
Glutamate Degradation X	4.57E-03	5.00E-01	*GLUD2*
Role of Oct4 in Mammalian Embryonic Stem Cell Pluripotency	5.01E-03	4.35E-02	*TP53,PHC3*
Lymphotoxin β Receptor Signaling	6.76E-03	3.70E-02	*MAP3K14,TRAF3*
NF-κB Signaling	7.24E-03	1.73E-02	*MAP3K14,TRAF3,NTRK1*
Induction of Apoptosis by HIV1	8.32E-03	3.33E-02	*TP53,MAP3K14*
CD40 Signaling	9.77E-03	3.08E-02	*MAP3K14,TRAF3*
Small Cell Lung Cancer Signaling	1.15E-02	2.82E-02	*TP53,TRAF3*
STAT3 Pathway	1.20E-02	2.74E-02	*NTRK1,SOCS2*
Arginine Biosynthesis IV	1.38E-02	1.67E-01	*GLUD2*
Aspartate Degradation II	1.58E-02	1.43E-01	*MDH2*
Altered T Cell and B Cell Signaling in Rheumatoid Arthritis	1.74E-02	2.27E-02	*MAP3K14,TRAF3*
Apoptosis Signaling	1.78E-02	2.25E-02	*TP53,MAP3K14*
Assembly of RNA Polymerase I Complex	2.04E-02	1.11E-01	*TAF1C*
p53 Signaling	2.14E-02	2.04E-02	*TP53,ST13*
Telomerase Signaling	2.14E-02	2.02E-02	*TP53,POT1*
Type I Diabetes Mellitus Signaling	2.63E-02	1.82E-02	*MAP3K14,SOCS2*
Type II Diabetes Mellitus Signaling	2.95E-02	1.71E-02	*MAP3K14,SOCS2*
Telomere Extension by Telomerase	3.39E-02	6.67E-02	*POT1*
Adipogenesis pathway	3.39E-02	1.57E-02	*TP53,FBXW7*
RAN Signaling	3.80E-02	5.88E-02	*KPNA2*
GADD45 Signaling	4.27E-02	5.26E-02	*TP53*

DNA damage-induced 14-3-3σ Signaling	4.27E-02	5.26E-02	*TP53*

Cocker spaniel + Golden retriever

Ingenuity Canonical Pathways	p-value	Ratio	Genes
Thyroid Cancer Signaling	1.17E-04	7.50E-02	*TP53,MYC,NTRK1*
Small Cell Lung Cancer Signaling	6.46E-04	4.23E-02	*TP53,MYC,TRAF3*
STAT3 Pathway	7.08E-04	4.11E-02	*MYC,NTRK1,SOCS2*
Telomerase Signaling	1.70E-03	3.03E-02	*TP53,MYC,POT1*
NGF Signaling	2.14E-03	2.80E-02	*TP53,MAP3K14,NTRK1*
TWEAK Signaling	2.95E-03	5.88E-02	*MAP3K14,TRAF3*
April Mediated Signaling	3.72E-03	5.26E-02	*MAP3K14,TRAF3*
Role of PKR in Interferon Induction and Antiviral Response	4.07E-03	5.00E-02	*TP53,TRAF3*
B Cell Activating Factor Signaling	4.07E-03	5.00E-02	*MAP3K14,TRAF3*
Melanoma Signaling	4.57E-03	4.76E-02	*TP53,MITF*
Role of Oct4 in Mammalian Embryonic Stem Cell Pluripotency	5.37E-03	4.35E-02	*TP53,PHC3*
Endometrial Cancer Signaling	6.92E-03	3.85E-02	*TP53,MYC*
Lymphotoxin β Receptor Signaling	7.41E-03	3.70E-02	*MAP3K14,TRAF3*
Acute Phase Response Signaling	7.59E-03	1.78E-02	*MAP3K14,SOCS2,HRG*
Wnt/β-catenin Signaling	7.59E-03	1.78E-02	*SOX17,TP53,MYC*
NF-κB Signaling	8.13E-03	1.73E-02	*MAP3K14,TRAF3,NTRK1*
Myc Mediated Apoptosis Signaling	8.51E-03	3.45E-02	*TP53,MYC*
Induction of Apoptosis by HIV1	9.12E-03	3.33E-02	*TP53,MAP3K14*
ERK5 Signaling	1.00E-02	3.17E-02	*MYC,NTRK1*
Cell Cycle: G1/S Checkpoint Regulation	1.02E-02	3.12E-02	*TP53,MYC*
CD40 Signaling	1.05E-02	3.08E-02	*MAP3K14,TRAF3*
Prolactin Signaling	1.32E-02	2.74E-02	*MYC,SOCS2*
Bladder Cancer Signaling	1.82E-02	2.30E-02	*TP53,MYC*
Altered T Cell and B Cell Signaling in Rheumatoid Arthritis	1.86E-02	2.27E-02	*MAP3K14,TRAF3*
RANK Signaling in Osteoclasts	1.86E-02	2.27E-02	*MAP3K14,MITF*
Apoptosis Signaling	1.91E-02	2.25E-02	*TP53,MAP3K14*
Chronic Myeloid Leukemia Signaling	2.09E-02	2.15E-02	*TP53,MYC*
Assembly of RNA Polymerase I Complex	2.14E-02	1.11E-01	*TAF1C*
Mouse Embryonic Stem Cell Pluripotency	2.19E-02	2.11E-02	*TP53,MYC*
p53 Signaling	2.29E-02	2.04E-02	*TP53,ST13*
Type I Diabetes Mellitus Signaling	2.82E-02	1.82E-02	*MAP3K14,SOCS2*
Type II Diabetes Mellitus Signaling	3.16E-02	1.71E-02	*MAP3K14,SOCS2*
p38 MAPK Signaling	3.16E-02	1.71E-02	*TP53,MYC*

Role of Macrophages, Fibroblasts and Endothelial Cells in Rheumatoid Arthritis	3.39E-02	1.01E-02	*MYC,MAP3K14,TRAF3*
Telomere Extension by Telomerase	3.55E-02	6.67E-02	*POT1*
Adipogenesis pathway	3.72E-02	1.57E-02	*TP53,FBXW7*
RAN Signaling	3.98E-02	5.88E-02	*KPNA2*
Aryl Hydrocarbon Receptor Signaling	4.37E-02	1.43E-02	*TP53,MYC*
GADD45 Signaling	4.47E-02	5.26E-02	*TP53*
DNA damage-induced 14-3-3σ Signaling	4.47E-02	5.26E-02	*TP53*
Glioblastoma Multiforme Signaling	4.79E-02	1.37E-02	*TP53,MYC*
