# Supplementary material for: Exome sequencing of lymphomas from three dog breeds reveals somatic mutation patterns reflecting genetic background
Source: Genome Res. 2015 Nov;25(11):1634–45. doi: 10.1101/gr.194449.115 (PMC4617960; doi:10.1101/gr.194449.115)
Supplement: Supplemental Material [file supp_gr.194449.115_Supp_Table5.pdf]

Supplementary Table 5. IPA-suggested canonical pathways ($p<0.05$) for significantly mutated genes from T-cell lymphomas from boxer and golden retriever

Boxer

Ingenuity Canonical Pathways	p-value	Ratio	Genes
Melanoma Signaling	6.31E-05	4.76E-02	*MAP2K1,PTEN*
Endometrial Cancer Signaling	9.77E-05	3.85E-02	*MAP2K1,PTEN*
ErbB2-ErbB3 Signaling	1.17E-04	3.51E-02	*MAP2K1,PTEN*
IL-17A Signaling in Airway Cells	1.48E-04	3.12E-02	*MAP2K1,PTEN*
Prostate Cancer Signaling	2.45E-04	2.44E-02	*MAP2K1,PTEN*
FAK Signaling	2.75E-04	2.30E-02	*MAP2K1,PTEN*
Neuregulin Signaling	2.82E-04	2.27E-02	*MAP2K1,PTEN*
Glioma Signaling	3.31E-04	2.11E-02	*MAP2K1,PTEN*
PTEN Signaling	5.01E-04	1.69E-02	*MAP2K1,PTEN*
PI3K/AKT Signaling	5.50E-04	1.63E-02	*MAP2K1,PTEN*
PI3K Signaling in B Lymphocytes	5.89E-04	1.56E-02	*MAP2K1,PTEN*
Ovarian Cancer Signaling	6.17E-04	1.53E-02	*MAP2K1,PTEN*
Insulin Receptor Signaling	6.46E-04	1.49E-02	*MAP2K1,PTEN*
Glioblastoma Multiforme Signaling	7.76E-04	1.37E-02	*MAP2K1,PTEN*
RAR Activation	1.12E-03	1.14E-02	*MAP2K1,PTEN*
B Cell Receptor Signaling	1.12E-03	1.14E-02	*MAP2K1,PTEN*
Sertoli Cell-Sertoli Cell Junction Signaling	1.15E-03	1.12E-02	*MAP2K1,PTEN*
Integrin Signaling	1.48E-03	9.90E-03	*MAP2K1,PTEN*
Protein Kinase A Signaling	5.25E-03	5.18E-03	*MAP2K1,PTEN*
D-myo-inositol (1,3,4)-trisphosphate Biosynthesis	5.62E-03	5.26E-02	*PTEN*
Superpathway of D-myo-inositol (1,4,5)-trisphosphate Metabolism	7.08E-03	4.17E-02	*PTEN*
4-1BB Signaling in T Lymphocytes	9.12E-03	3.23E-02	*MAP2K1*
Oncostatin M Signaling	1.00E-02	2.94E-02	*MAP2K1*
Thyroid Cancer Signaling	1.20E-02	2.50E-02	*MAP2K1*
UVC-Induced MAPK Signaling	1.26E-02	2.38E-02	*MAP2K1*
Role of IL-17F in Allergic Inflammatory Airway Diseases	1.32E-02	2.27E-02	*MAP2K1*
CD27 Signaling in Lymphocytes	1.55E-02	1.92E-02	*MAP2K1*
CNTF Signaling	1.55E-02	1.92E-02	*MAP2K1*
UVB-Induced MAPK Signaling	1.58E-02	1.89E-02	*MAP2K1*
IL-2 Signaling	1.58E-02	1.89E-02	*MAP2K1*
Role of IL-17A in Arthritis	1.58E-02	1.85E-02	*MAP2K1*
Thrombopoietin Signaling	1.62E-02	1.82E-02	*MAP2K1*
EGF Signaling	1.66E-02	1.79E-02	*MAP2K1*
ErbB4 Signaling	1.78E-02	1.67E-02	*MAP2K1*
GM-CSF Signaling	1.82E-02	1.61E-02	*MAP2K1*
Antiproliferative Role of Somatostatin Receptor 2	1.86E-02	1.59E-02	*MAP2K1*
Pyridoxal 5'-phosphate Salvage Pathway	1.91E-02	1.56E-02	*MAP2K1*

CD40 Signaling	1.91E-02	1.54E-02	*MAP2K1*
Non-Small Cell Lung Cancer Signaling	1.91E-02	1.54E-02	*MAP2K1*
Hypoxia Signaling in the Cardiovascular System	1.91E-02	1.54E-02	*PTEN*
IL-15 Signaling	1.95E-02	1.52E-02	*MAP2K1*
Role of PI3K/AKT Signaling in the Pathogenesis of Influenza	1.95E-02	1.52E-02	*MAP2K1*
Erythropoietin Signaling	2.00E-02	1.49E-02	*MAP2K1*
Neurotrophin/TRK Signaling	2.00E-02	1.49E-02	*MAP2K1*
Role of MAPK Signaling in the Pathogenesis of Influenza	2.00E-02	1.47E-02	*MAP2K1*
GDNF Family Ligand-Receptor Interactions	2.00E-02	1.47E-02	*MAP2K1*
Melatonin Signaling	2.09E-02	1.43E-02	*MAP2K1*
IL-3 Signaling	2.09E-02	1.41E-02	*MAP2K1*
Renal Cell Carcinoma Signaling	2.09E-02	1.41E-02	*MAP2K1*
Small Cell Lung Cancer Signaling	2.09E-02	1.41E-02	*PTEN*
Chemokine Signaling	2.09E-02	1.41E-02	*MAP2K1*
IL-17 Signaling	2.14E-02	1.39E-02	*MAP2K1*
JAK/Stat Signaling	2.14E-02	1.39E-02	*MAP2K1*
LPS-stimulated MAPK Signaling	2.14E-02	1.37E-02	*MAP2K1*
Prolactin Signaling	2.14E-02	1.37E-02	*MAP2K1*
STAT3 Pathway	2.14E-02	1.37E-02	*MAP2K1*
FLT3 Signaling in Hematopoietic Progenitor Cells	2.19E-02	1.35E-02	*MAP2K1*
Leptin Signaling in Obesity	2.19E-02	1.35E-02	*MAP2K1*
TREM1 Signaling	2.24E-02	1.33E-02	*NLRP14*
VEGF Family Ligand-Receptor Interactions	2.24E-02	1.32E-02	*MAP2K1*
BMP signaling pathway	2.24E-02	1.32E-02	*MAP2K1*
Acute Myeloid Leukemia Signaling	2.29E-02	1.30E-02	*MAP2K1*
PDGF Signaling	2.29E-02	1.30E-02	*MAP2K1*
Regulation of IL-2 Expression in Activated and Anergic T Lymphocytes	2.34E-02	1.27E-02	*MAP2K1*
Ceramide Signaling	2.34E-02	1.25E-02	*MAP2K1*
Melanocyte Development and Pigmentation Signaling	2.45E-02	1.19E-02	*MAP2K1*
FGF Signaling	2.51E-02	1.18E-02	*MAP2K1*
ErbB Signaling	2.51E-02	1.16E-02	*MAP2K1*
α-Adrenergic Signaling	2.57E-02	1.15E-02	*MAP2K1*
Bladder Cancer Signaling	2.57E-02	1.15E-02	*MAP2K1*
TGF-β Signaling	2.57E-02	1.15E-02	*MAP2K1*
RANK Signaling in Osteoclasts	2.57E-02	1.14E-02	*MAP2K1*
PAK Signaling	2.63E-02	1.12E-02	*MAP2K1*
Apoptosis Signaling	2.63E-02	1.12E-02	*MAP2K1*
VEGF Signaling	2.69E-02	1.10E-02	*MAP2K1*

Fcγ Receptor-mediated Phagocytosis in Macrophages and Monocytes	2.75E-02	1.08E-02	*PTEN*
Chronic Myeloid Leukemia Signaling	2.75E-02	1.08E-02	*MAP2K1*
PPAR Signaling	2.75E-02	1.06E-02	*MAP2K1*
Mouse Embryonic Stem Cell Pluripotency	2.82E-02	1.05E-02	*MAP2K1*
Salvage Pathways of Pyrimidine Ribonucleotides	2.82E-02	1.05E-02	*MAP2K1*
IGF-1 Signaling	2.88E-02	1.03E-02	*MAP2K1*
T Cell Receptor Signaling	2.88E-02	1.03E-02	*MAP2K1*
p53 Signaling	2.88E-02	1.02E-02	*PTEN*
CDK5 Signaling	2.95E-02	1.01E-02	*MAP2K1*
Telomerase Signaling	2.95E-02	1.01E-02	*MAP2K1*
Nitric Oxide Signaling in the Cardiovascular System	2.95E-02	1.01E-02	*MAP2K1*
Cholecystokinin/Gastrin-mediated Signaling	2.95E-02	9.90E-03	*MAP2K1*
Rac Signaling	3.09E-02	9.62E-03	*MAP2K1*
HGF Signaling	3.09E-02	9.52E-03	*MAP2K1*
Pancreatic Adenocarcinoma Signaling	3.09E-02	9.43E-03	*MAP2K1*
NGF Signaling	3.16E-02	9.35E-03	*MAP2K1*
fMLP Signaling in Neutrophils	3.16E-02	9.26E-03	*MAP2K1*
iCOS-iCOSL Signaling in T Helper Cells	3.16E-02	9.26E-03	*PTEN*
Fc Epsilon RI Signaling	3.24E-02	9.17E-03	*MAP2K1*
Renin-Angiotensin Signaling	3.24E-02	9.17E-03	*MAP2K1*
Gαs Signaling	3.24E-02	9.17E-03	*MAP2K1*
Natural Killer Cell Signaling	3.24E-02	9.09E-03	*MAP2K1*
Role of Tissue Factor in Cancer	3.24E-02	9.09E-03	*PTEN*
Corticotropin Releasing Hormone Signaling	3.24E-02	9.01E-03	*MAP2K1*
Role of NANOG in Mammalian Embryonic Stem Cell Pluripotency	3.24E-02	9.01E-03	*MAP2K1*
Hereditary Breast Cancer Signaling	3.39E-02	8.70E-03	*PTEN*
IL-6 Signaling	3.39E-02	8.62E-03	*MAP2K1*
14-3-3-mediated Signaling	3.47E-02	8.55E-03	*MAP2K1*
CCR3 Signaling in Eosinophils	3.47E-02	8.55E-03	*MAP2K1*
Gα12/13 Signaling	3.47E-02	8.55E-03	*MAP2K1*
CD28 Signaling in T Helper Cells	3.47E-02	8.47E-03	*MAP2K1*
Synaptic Long Term Potentiation	3.47E-02	8.40E-03	*MAP2K1*
p70S6K Signaling	3.47E-02	8.40E-03	*MAP2K1*
P2Y Purigenic Receptor Signaling Pathway	3.47E-02	8.40E-03	*MAP2K1*
HMGB1 Signaling	3.55E-02	8.33E-03	*MAP2K1*
D-myo-inositol (1,4,5,6)-Tetrakisphosphate Biosynthesis	3.72E-02	7.87E-03	*PTEN*
D-myo-inositol (3,4,5,6)-tetrakisphosphate Biosynthesis	3.72E-02	7.87E-03	*PTEN*

Estrogen Receptor Signaling	3.72E-02	7.87E-03	*MAP2K1*
GNRH Signaling	3.80E-02	7.75E-03	*MAP2K1*
IL-12 Signaling and Production in Macrophages	3.98E-02	7.41E-03	*MAP2K1*
Relaxin Signaling	3.98E-02	7.41E-03	*MAP2K1*
Synaptic Long Term Depression	4.17E-02	7.09E-03	*MAP2K1*
D-myo-inositol-5-phosphate Metabolism	4.17E-02	6.99E-03	*PTEN*
3-phosphoinositide Degradation	4.27E-02	6.94E-03	*PTEN*
Regulation of eIF4 and p70S6K Signaling	4.27E-02	6.85E-03	*MAP2K1*
Epithelial Adherens Junction Signaling	4.27E-02	6.85E-03	*PTEN*
Gαq Signaling	4.27E-02	6.80E-03	*MAP2K1*
CXCR4 Signaling	4.47E-02	6.58E-03	*MAP2K1*
Aldosterone Signaling in Epithelial Cells	4.47E-02	6.58E-03	*MAP2K1*
Gap Junction Signaling	4.57E-02	6.45E-03	*MAP2K1*
3-phosphoinositide Biosynthesis	4.57E-02	6.45E-03	*PTEN*
Germ Cell-Sertoli Cell Junction Signaling	4.68E-02	6.25E-03	*MAP2K1*
Tight Junction Signaling	4.90E-02	5.99E-03	*PTEN*
Acute Phase Response Signaling	4.90E-02	5.92E-03	*MAP2K1*

Golden retriever

Ingenuity Canonical Pathways	p-value	Ratio	Genes
Glutamate Biosynthesis II	1.70E-03	5.00E-01	*GLUD2*
Glutamate Degradation X	1.70E-03	5.00E-01	*GLUD2*
Oxidative Phosphorylation	3.80E-03	1.83E-02	*ATP5H,COX8A*
Arginine Biosynthesis IV	5.01E-03	1.67E-01	*GLUD2*
Mitochondrial Dysfunction	8.91E-03	1.17E-02	*ATP5H,COX8A*
EIF2 Signaling	1.05E-02	1.08E-02	*RPL11,RPL23A*
Leukotriene Biosynthesis	1.26E-02	6.67E-02	*LTA4H*
Pyrimidine Deoxyribonucleotides De Novo Biosynthesis I	1.86E-02	4.55E-02	*NME7*
Protein Ubiquitination Pathway	1.91E-02	7.84E-03	*FBXW7,PSMA1*
Role of JAK1, JAK2 and TYK2 in Interferon Signaling	2.00E-02	4.17E-02	*PTPN6*
Pyrimidine Ribonucleotides Interconversion	2.24E-02	3.70E-02	*NME7*
Pyrimidine Ribonucleotides De Novo Biosynthesis	2.40E-02	3.45E-02	*NME7*
Role of JAK2 in Hormone-like Cytokine Signaling	2.95E-02	2.86E-02	*PTPN6*
