# Supplementary material for: Exome sequencing of lymphomas from three dog breeds reveals somatic mutation patterns reflecting genetic background
Source: Genome Res. 2015 Nov;25(11):1634–45. doi: 10.1101/gr.194449.115 (PMC4617960; doi:10.1101/gr.194449.115)
Supplement: Supplemental Material [file supp_gr.194449.115_Supp_Table6.pdf]

Supplementary Table 6. All somatic copy number alterations

Descriptor	Peak Limits	Region Limits	q values	Residual q values after removing segments shared with higher peaks
8q11.2	Chr8:2869427-2882658	Chr8:2343602-2943145	7.53E-22	2.98E-19
8q33.3	Chr8:72990670-73028555	Chr8:72851488-73956025	4.03E-20	8.14E-15
26q23	Chr26:27585064-27602759	Chr26:25572035-27700576	3.52E-13	3.52E-13
27q22.1	Chr27:35640457-35649322	Chr27:24720737-39830871	1.01E-07	1.01E-07
11q15	Chr11:41264940-41305867	Chr11:40303074-49723781	7.27E-09	1.81E-07
16q12	Chr16:6774247-6793678	Chr16:6766358-6907394	3.37E-09	3.15E-07
13q21.1	Chr13:37810498-37816495	Chr13:35456406-39018634	7.77E-06	7.77E-06
26q23	Chr26:27303323-27354974	Chr26:26174995-27626790	2.48E-05	2.48E-05
18q12	Chr18:11622188-11700181	Chr18:11622188-11730475	8.94E-05	8.94E-05
6q21.3	Chr6:38834153-38845543	Chr6:38574472-40324490	0.00025591	0.00025591
8q33.3	Chr8:72851488-72862981	Chr8:71697654-73272242	0.00025591	0.00026972
24q25	Chr24:47029779-47110403	Chr24:46720516-50000000	0.00062758	0.00062758
24q25	Chr24:44492687-44530980	Chr24:44475705-44573167	0.00089461	0.00089461
20q17	Chr20:57364803-57378205	Chr20:55670570-61000000	0.0012483	0.0012483
18q22.1	Chr18:25723457-25727487	Chr18:25232922-25865176	0.0016936	0.0016936
31q15.1	Chr31:29912719-30091933	Chr31:29863672-30120133	0.00047459	0.0016936
10q14	Chr10:17021157-17029882	Chr10:16620762-17127285	0.0017365	0.0017365
28q18	Chr28:40745394-40855815	Chr28:38755101-41088858	0.0017365	0.0017365
32q11.2	Chr32:13499741-14258756	Chr32:13066103-17172641	0.0019547	0.0019547
31q15.3	Chr31:39439925-39463350	Chr31:35259486-42000000	0.00025591	0.0024885
3q35.2	Chr3:91475161-91555106	Chr3:91322559-91823488	0.0030069	0.0030069
17q15	Chr17:37664976-37729565	Chr17:37512670-37898311	1.86E-07	0.0030229
34q15	Chr34:30298915-30320051	Chr34:30071537-30603599	0.0031191	0.0031191
25q24	Chr25:51593798-54000000	Chr25:50270659-54000000	0.0033599	0.0033599
14q13	Chr14:17613375-17642595	Chr14:17539908-17699298	0.0015803	0.0035972

17q21	Chr17:52543607-52648062	Chr17:52523852-52711573	0.0013175	0.0044557
8q33.3	Chr8:73830076-74052199	Chr8:73295440-76000000	0.00033104	0.004698
6q21.3	Chr6:40654674-40717881	Chr6:40654674-40730284	0.0050135	0.0050135
Xp22.3	ChrX:1092731-1107729	ChrX:1-3910135	0.0091611	0.0091611
22q12.3	Chr22:23163090-24007496	Chr22:22779370-25716401	0.010832	0.010832
2q35	Chr2:84567106-84649103	Chr2:84433804-84813166	0.011255	0.011255
38q11	Chr38:3655873-4906339	Chr38:3487306-5911768	0.0024689	0.011255
23q22	Chr23:32822241-33025550	Chr23:32814487-33295131	0.012074	0.012074
38q12	Chr38:8143822-8306862	Chr38:2878996-13430300	0.0031191	0.012312
4q33	Chr4:71601230-71646700	Chr4:71594461-71682197	0.013096	0.013096
14q11.1	Chr14:734998-773001	Chr14:680867-820781	0.015894	0.015894
19q12	Chr19:7096830-7490663	Chr19:6483792-7878842	0.016902	0.016902
25q21.1	Chr25:18647866-18684799	Chr25:18620335-18728671	0.017691	0.017691
30q14.3	Chr30:26692677-26715373	Chr30:26623101-27262693	0.019567	0.019567
7q11	Chr7:860370-861423	Chr7:725821-2244264	0.019692	0.019692
Xq21.1	ChrX:70256798-70494237	ChrX:69831751-70886627	0.021188	0.021188
30q15.2	Chr30:38448916-38513654	Chr30:38410113-38847301	0.021737	0.021737
3q21.3	Chr3:35781900-35830801	Chr3:35376390-35853242	0.023093	0.023093
20q12	Chr20:15918737-16174743	Chr20:15372283-17177158	0.023093	0.023093
29q22	Chr29:32866440-33053913	Chr29:32714879-33584560	0.023093	0.023093
4q21	Chr4:27338191-27688198	Chr4:27253140-27691185	0.0071576	0.023778
33q15.3	Chr33:31118217-31194881	Chr33:30724298-31228353	0.025857	0.025857
22q22	Chr22:41752310-42063443	Chr22:41752310-42507222	0.027645	0.027645
36q14	Chr36:21088926-21223646	Chr36:21086716-21316659	0.027893	0.027893
21q21	Chr21:20931557-20948030	Chr21:20931557-21101506	0.030164	0.030164
15q21	Chr15:22859792-22925932	Chr15:22744791-23002496	0.032707	0.032707
11q15	Chr11:40697160-40778780	Chr11:40587120-40807595	0.03435	0.03435
12q11	Chr12:2630951-2648769	Chr12:2630597-2652858	0.03435	0.03435
6q23.3	Chr6:56645856-56680529	Chr6:56556364-56763810	0.037865	0.037865
7q24	Chr7:69367517-69470236	Chr7:69290110-69522659	0.037865	0.037865

16q11	Chr16:1040745-1116146	Chr16:620520-1540168	0.015894	0.039302
10q21	Chr10:20660305-20696690	Chr10:20640796-20710067	0.041045	0.041045
33q15.3	Chr33:30665189-30692830	Chr33:30522061-30702619	0.043598	0.043598
38q15.2	Chr38:23205189-23230555	Chr38:23176452-23348400	0.00011167	0.043598
37q16	Chr37:26062437-26075328	Chr37:25917818-26145822	0.044153	0.044153
2q12.1	Chr2:4125467-4175081	Chr2:3750476-4277934	0.05131	0.05131
35q15	Chr35:23978673-24033701	Chr35:23974356-24035166	0.05131	0.05131
13q22.1	Chr13:58733448-58848893	Chr13:58722105-58857340	0.056497	0.056497
31q12	Chr31:5743040-5875968	Chr31:3006824-7459338	0.056497	0.056497
36q14	Chr36:22271665-22275238	Chr36:22256781-22310276	0.059035	0.059035
21q24.3	Chr21:48498367-48714917	Chr21:48497120-48818064	0.061125	0.061125
15q12	Chr15:7167388-7176134	Chr15:7002787-8119769	0.027645	0.062188
5q32	Chr5:58061505-58067752	Chr5:58061505-58076016	0.015171	0.069545
17q15	Chr17:37680900-37885734	Chr17:37663281-37919796	0.069545	0.069545
1q12	Chr1:14634373-14750325	Chr1:14598796-15184745	0.072649	0.072649
12q14	Chr12:22623074-22858972	Chr12:22617496-23201180	0.072649	0.072649
9q11.1	Chr9:1-105569	Chr9:1-542733	0.073672	0.078069
38q15.2	Chr38:23533944-23682209	Chr38:23490623-26000000	0.078069	0.084238
17q11.1	Chr17:480259-908364	Chr17:452624-1312728	0.087113	0.087113
17q11.1	Chr17:785549-793522	Chr17:484882-855766	0.035496	0.087488
16q14	Chr16:25534254-25810123	Chr16:25532537-26032741	0.035496	0.095502
9q12	Chr9:15667778-15732575	Chr9:15602494-15918909	0.10331	0.10331
1q37	Chr1:116498108-116513465	Chr1:116376480-116553316	0.1133	0.1133
29q11	Chr29:1316620-1668539	Chr29:1316620-2648115	0.1133	0.1133
19q21	Chr19:20921074-21387426	Chr19:20816724-21551814	0.11611	0.11611
Xq27	ChrX:117533004-117640474	ChrX:117532309-117710833	0.078069	0.11611
15q11.2	Chr15:4578939-4604406	Chr15:4578939-4605711	0.056855	0.12379
5q24	Chr5:47506287-47551444	Chr5:47496346-47578506	0.1434	0.1434
28q12	Chr28:9573472-9592296	Chr28:9573472-9639007	0.1434	0.1434
27q11	Chr27:1018760-1021963	Chr27:1016319-1039695	0.1133	0.14807

Xq21.1	ChrX:62059491-62810656	ChrX:62059491-64922672	0.14807	0.14807
Xq27	ChrX:121554736-121799777	ChrX:121535138-122206773	0.11611	0.14807
9q25	Chr9:55989911-56041217	Chr9:55978509-56084436	0.062188	0.16951
16q25.2	Chr16:58935686-58941613	Chr16:58935686-59045083	0.046004	0.16951
4q32	Chr4:67163444-67651590	Chr4:67163444-67673240	0.17837	0.19336
5q21	Chr5:32884479-32902874	Chr5:32884479-32904791	0.13898	0.19336
35q12	Chr35:3471260-3561795	Chr35:3471260-3665572	0.19336	0.19336
12q21.2	Chr12:35649706-35660063	Chr12:35649706-35665465	0.20609	0.20609
34q12	Chr34:11432627-11450687	Chr34:11432627-11452765	0.20609	0.20609
14q22	Chr14:55652984-55688774	Chr14:55652984-55780630	0.17348	0.2076
23q13	Chr23:11786085-11815528	Chr23:11786085-11816873	0.21735	0.21735
37q14	Chr37:18329846-18398779	Chr37:18329846-18405541	0.21828	0.21828
11q11	Chr11:6467671-7070399	Chr11:6413996-7975342	0.023093	0.23405
Xq27	ChrX:122052459-122069620	ChrX:122052459-122070465	0.24438	0.24438
27q22.1	Chr27:35133080-35385921	Chr27:35133080-35706656	0.041235	0.30741
27q11	Chr27:2929170-2950008	Chr27:2850850-3077645	0.056855	0.45174
1q25.1	Chr1:72866577-73377883	Chr1:72866577-73402282	0.083172	1
4q21	Chr4:27338191-27679960	Chr4:27253140-27691185	0.0071576	1
22q22	Chr22:41752310-42063443	Chr22:41752310-42507222	0.027645	1
