# Supplementary material for: Exome sequencing of lymphomas from three dog breeds reveals somatic mutation patterns reflecting genetic background
Source: Genome Res. 2015 Nov;25(11):1634–45. doi: 10.1101/gr.194449.115 (PMC4617960; doi:10.1101/gr.194449.115)
Supplement: Supplemental Material [file supp_gr.194449.115_Supp_Table7.pdf]

**Supplementary Table 7.** Networks suggested by IPA for significantly mutated genes from B-cell lymphomas from cocker spaniel and golden retriever

**Cocker spaniel**

| Network ID | Molecules in Network | Score | Focus Molecules | Top Diseases and Functions |
|---|---|---|---|---|
| 1 | Akt,APP,CAPRIN1,caspase,DDX3X,EDA,EIF1AX,FAM90A1, FFAR2,GAPDHS,GNPTAB,GPM6B,GYG2,Igm,INPP4B,MITF, MYC,P38 MAPK,PABPN1,PLBD1,POT1,PPFIBP2,PSMA1, RAD51C,SLC19A2,ST3GAL6,TBKBP1,TMCC2,TNFRSF13C, TRAF3,TRAF4,TRAF1-TRAF2-TRAF3,UBL3,USP48,Vegf | 20 | 7 | Cell Morphology, Cancer, Cell Death and Survival |

**Golden retriever**

| Network ID | Molecules in Network | Score | Focus Molecules | Top Diseases and Functions |
|---|---|---|---|---|
| 1 | 26s Proteasome,Actin,Ap1,DDX3X,EEF1A1,EEF1A2,ERG,ERK1/2, GLUD1,HBA1/HBA2,Hsp70,IFN Beta,IgG2a,Igm,Ikb, Immunoglobulin,Insulin,KLRC4-KLRK1/KLRK1,MAP3K14, MDH2,NTRK1,p70 S6k,p85 (pik3r),PDIA6,PDK1,PI3K (complex),PKD1,PNRC1,Ras,RPL22,RPL23A,Sos,thyroid hormone receptor,TRAF3,XRN2 | 43 | 18 | Cellular Development, Tissue Development, Protein Synthesis |
| 2 | ANKMY2,APP,ATP5H,BAI3,C12orf5,CFAP58,CPPED1,CWC15, CYP4F11,DUS2,EMR2,ETS1,FAM90A1,GPR52,HOMEZ,IL6, ISYNA1,KIAA1191,KNOP1,LINS,MAPRE1,PHC3,PHYHD1, PLBD1,POT1,PROSER2,SUMO2,SYAP1,UBC,UBL3,USP17L2 (includes others),WDR17,ZBED2,ZDHHC2,ZNF486 | 30 | 14 | Cell-To-Cell Signaling and Interaction, Tissue Development, Cellular Compromise |

| | | | | |
|---|---|---|---|---|
| 3 | Akt,BRK1,caspase,CD3,Ck2,Creb,DR4/5,ERK,FBXW7,FKBP3, Histone h3,HNRNPA1L2,Hsp90,Jnk,Jnk dimer,KPNA2,miR-17-3p (and other miRNAs w/seed CUGCAGU),miR-191-5p (and other miRNAs w/seed AACGGAA),MIR155HG,NFkB (complex),P38 MAPK,Pkc(s),PRELID1,PSMA1,RNA polymerase II,SETD2,SOCS2,SOX17,ST13,TAF1C,TP53,TRAF1-TRAF2-TRAF3,TRIAP1,Vegf,ZNF385A | 24 | 12 | Cancer, Cell Cycle, Cell Morphology |
| 4 | AICAR,APH1A,APLP2,BUB3,CD47,DOK2,DOK6,DYRK1A,E2F2, EGFR,ELAVL1,EPHA2,FKBP1A,HCFC1,HTATIP2,IGF2BP3, ISCA1,mir-15,MYBL2,PDCD6IP,PEX19,PHLDA1,PPP2R2A, PPP6C,RALA,RHOB,RNF41,RPN1,SCAMP1,SERPINE2,SGPL1, SH2D1A,TUBB3,UBE2L3,YES1 | 5 | 3 | Cancer, Cell Cycle, Infectious Disease |

**Combined dataset (cocker spaniel + golden retriever)**

| Network ID | Molecules in Network | Score | Focus Molecules | Top Diseases and Functions |
|---|---|---|---|---|
| 1 | 26s Proteasome,60S ribosomal subunit,Akt,caspase,Cg,Ck2, DDX3X,ERG,FBXW7,Hdac,HISTONE,Histone h3,HNRNPA1L2, HRG,Hsp70,Hsp90,Immunoglobulin,KPNA2,MAP3K14,MYC, NFkB (complex),POT1,PPP6C,PSMA1,RNA polymerase II, RPL22,RPL23A,SETD2,ST13,TAF1C,TAF1L,TP53,Ubiquitin, USP17L2 (includes others),XRN2 | 45 | 20 | Cancer, Hematological Disease, Immunological Disease |
| 2 | Ap1,BRK1,Cbp/p300,CD3,Creb,Cyclin E,EEF1A2,ERK1/2, FKBP3,Growth hormone,HBA1/HBA2,IFN Beta,Ige,IgG, IgG2a,Igm,Ikb,IL1,IL12 (complex),Interferon alpha,KLRC4-KLRK1/KLRK1,Mek,MITF,NTRK1,OGT,p85 (pik3r),PDK1, PNRC1,Ras,SOCS2,Sos,SOX17,TCR,thyroid hormone receptor,TRAF3 | 28 | 13 | Cellular Movement, Cellular Development, Tissue Development |

| | | | | |
|---|---|---|---|---|
| 3 | APBB2,APOBEC3C,APP,ARID1B,ATL3,ATP5H,BAI3,C12orf5, CWC15,DOK6,DUS2,EGFR,ELAVL1,EMR2,FAM90A1,GPD1L, ISCA1,LETMD1,LINGO1,LINS,MAPRE1,MBNL2,MIS18BP1, MPI,MPV17L,MRPL24,PHC3,PLBD1,SLC38A5,SUMF2, TMED7,TMEM127,UBC,UBL3,ZDHHC2 | 27 | 13 | Developmental Disorder, Hereditary Disorder, Neurological Disease |
| 4 | ARHGAP1,Cg,DHCR24,ELK3,ELK4,ERK,FGFBP1,GAS6,GPR34, Gsk3,indican,iodine,Jnk,K Channel,KNOP1,LRRN3,MAP3K4, Mapk,MAPK4,mir-101,miR-199a-3p (and other miRNAs w/seed CAGUAGU),MKK3/6,NCF4,P38 MAPK,PADI2,PI3K (complex),Pkc(s),PKD1,PODXL,Rac,TLR6,TRAF1-TRAF2-TRAF3,TRIO,Vegf,VRK2 | 5 | 3 | Cellular Development, Cellular Growth and Proliferation, Renal Proliferation |
