# Supplementary material for: Exome sequencing of lymphomas from three dog breeds reveals somatic mutation patterns reflecting genetic background
Source: Genome Res. 2015 Nov;25(11):1634–45. doi: 10.1101/gr.194449.115 (PMC4617960; doi:10.1101/gr.194449.115)
Supplement: Supplemental Material [file supp_gr.194449.115_Supp_Table8.pdf]

Supplementary Table 8. Functional annotation charts from DAVID analysis of top significantly mutated genes (if more than 15 significantly mutated genes, only the top 15 genes were used since DAVID does not take p-values)

Cocker spaniel B-cell lymphoma

	Category	Term	Description	% input genes implicated	p-value	List Total	Pop Hits	Pop Total
1	GOTERM_BP_FAT	GO0010604	positive regulation of macromolecule metabolic process	57.1	9.66E-04	5	857	13528
2	GOTERM_BP_FAT	GO0032204	regulation of telomere maintenance	28.6	3.54E-03	5	12	13528
3	GOTERM_BP_FAT	GO0043085	positive regulation of catalytic activity	42.9	8.40E-03	5	520	13528
4	GOTERM_BP_FAT	GO0033044	regulation of chromosome organization	28.6	8.84E-03	5	30	13528
5	GOTERM_BP_FAT	GO0044093	positive regulation of molecular function	42.9	1.06E-02	5	586	13528
6	GOTERM_BP_FAT	GO0045935	positive regulation of nucleobase, nucleoside, nucleotide and nucleic acid metabolic process	42.9	1.20E-02	5	624	13528
7	GOTERM_BP_FAT	GO0051173	positive regulation of nitrogen compound metabolic process	42.9	1.27E-02	5	644	13528
8	GOTERM_BP_FAT	GO0010557	positive regulation of macromolecule biosynthetic process	42.9	1.31E-02	5	654	13528
9	GOTERM_CC_FAT	GO0005654	nucleoplasm	42.9	1.36E-02	4	882	12782
10	GOTERM_BP_FAT	GO0031328	positive regulation of cellular biosynthetic process	42.9	1.43E-02	5	685	13528
11	SP_PIR_KEYWORDS	dna-binding		57.1	1.46E-02	7	1868	19235
12	GOTERM_BP_FAT	GO0009891	positive regulation of biosynthetic process	42.9	1.48E-02	5	695	13528
13	GOTERM_BP_FAT	GO0010605	negative regulation of macromolecular metabolic process	42.9	1.64E-02	5	734	13528

14	GOTERM_BP_FAT	GO0043473	pigmentation	28.6	1.73E-02	5	59	13528
15	GOTERM_BP_FAT	GO0042981	regulation of apoptosis	42.9	1.95E-02	5	804	13528
16	GOTERM_BP_FAT	GO0043067	regulation of programmed cell death	42.9	1.99E-02	5	812	13528
17	GOTERM_BP_FAT	GO0010941	regulation of cell death	42.9	2.00E-02	5	815	13528
18	KEGG_PATHWAY	hsa05200	Pathways in cancer	42.9	2.28E-02	5	328	5085
19	SP_PIR_KEYWORDS	nucleus		71.4	2.49E-02	7	4283	19235
20	GOTERM_MF_FAT	GO0043565	sequence-specific DNA binding	42.9	2.89E-02	7	607	12983
21	GOTERM_BP_FAT	GO0051052	regulation of Dna metabolic process	28.6	3.33E-02	5	114	13528
22	GOTERM_BP_FAT	GO0032844	regulation of homeostatic process	28.6	3.33E-02	5	114	13528
23	UP_SEQ_FEATURE	domain	Leucine-zipper	28.6	3.40E-02	7	110	19113
24	UP_SEQ_FEATURE	domain	Helix-loop-helix motif	28.6	3.53E-02	7	114	19113
25	GOTERM_CC_FAT	GO0031981	nuclear lumen	42.9	3.57E-02	4	1450	12782
26	GOTERM_CC_FAT	GO0016604	nuclear body	28.6	3.89E-02	4	168	12782
27	INTERPRO	IPR001092	Basic helix-loop-helix dimerisation region bHLH	28.6	4.04E-02	7	114	16659
28	SMART	SM00353	HLH	28.6	4.93E-02	5	114	9079
29	UP_SEQ_FEATURE	DNA-binding region	Basic motif	28.6	4.95E-02	7	161	19113
30	GOTERM_BP_FAT	GO0051345	positive regulation of hydrolase activity	28.6	5.19E-02	5	179	13528
31	GOTERM_CC_FAT	GO0070013	intracellular organelle lumen	42.9	5.27E-02	4	1779	12782
32	KEGG_PATHWAY	hsa04622	RIG-I-like receptor signaling pathway	28.6	5.47E-02	5	71	5085
33	GOTERM_CC_FAT	GO0043233	organelle lumen	42.9	5.50E-02	4	1820	12782
34	GOTERM_CC_FAT	GO0031974	membrane-enclosed lumen	42.9	5.71E-02	4	1856	12782
35	GOTERM_BP_FAT	GO0033043	regulation of organelle organization	28.6	6.26E-02	5	217	13528
36	KEGG_PATHWAY	hsa05222	Small cell lung cancer	28.6	6.45E-02	5	84	5085
37	GOTERM_MF_FAT	GO0043566	structure-specific DNA binding	28.6	6.52E-02	7	145	12983

38	GOTERM_BP_FAT	GO0007423	sensory organ development	28.6	6.60E-02	5	229	13528
39	GOTERM_MF_FAT	GO0003677	DNA binding	57.1	7.53E-02	7	2331	12983
40	GOTERM_BP_FAT	GO0043086	negative regulation of catalytic activity	28.6	7.94E-02	5	277	13528
41	GOTERM_BP_FAT	GO0006917	induction of apoptosis	28.6	9.13E-02	5	320	13528
42	GOTERM_BP_FAT	GO0012502	induction of programmed cell death	28.6	9.16E-02	5	321	13528
43	GOTERM_BP_FAT	GO0044092	negative regulation of molecular function	28.6	9.52E-02	5	334	13528
44	SP_PIR_KEYWORDS	isopeptide bond		28.6	9.55E-02	7	319	19235
45	GOTERM_BP_FAT	GO0051336	regulation of hydrolase activity	28.6	9.60E-02	5	337	13528

Cocker spaniel B-cell lymphoma, continued

	Category	Term	Fold Enrichment	Bonferroni	Benjamini	FDR	Genes
1	GOTERM_BP_FAT	GO0010604	12.63	0.19	0.19	1.22	ENSG00000136997,ENSG00000187098,ENSG00000129084,ENSG00000128513
2	GOTERM_BP_FAT	GO0032204	450.93	0.55	0.33	4.42	ENSG00000136997,ENSG00000128513
3	GOTERM_BP_FAT	GO0043085	15.61	0.85	0.46	10.20	ENSG00000136997,ENSG00000129084,ENSG00000128513
4	GOTERM_BP_FAT	GO0033044	180.37	0.86	0.39	10.70	ENSG00000136997,ENSG00000128513
5	GOTERM_BP_FAT	GO0044093	13.85	0.91	0.38	12.71	ENSG00000136997,ENSG00000129084,ENSG00000128513
6	GOTERM_BP_FAT	GO0045935	13.01	0.93	0.36	14.24	ENSG00000136997,ENSG00000187098,ENSG00000128513
7	GOTERM_BP_FAT	GO0051173	12.60	0.94	0.33	15.07	ENSG00000136997,ENSG00000187098,ENSG00000128513

8	GOTERM_BP_FAT	GO0010557	12.41	0.95	0.31	15.49	ENSG00000136997,ENSG00000187098,ENSG00000128513
9	GOTERM_CC_FAT	GO0005654	10.87	0.33	0.33	10.93	ENSG00000215301,ENSG00000136997,ENSG00000128513
10	GOTERM_BP_FAT	GO0031328	11.85	0.96	0.30	16.83	ENSG00000136997,ENSG00000187098,ENSG00000128513
11	SP_PIR_KEYWORDS	dna-binding	5.88	0.44	0.44	12.59	ENSG00000215301,ENSG00000136997,ENSG00000187098,ENSG00000128513
12	GOTERM_BP_FAT	GO0009891	11.68	0.96	0.28	17.26	ENSG00000136997,ENSG00000187098,ENSG00000128513
13	GOTERM_BP_FAT	GO0010605	11.06	0.97	0.28	19.00	ENSG00000136997,ENSG00000129084,ENSG00000128513
14	GOTERM_BP_FAT	GO0043473	91.72	0.98	0.28	19.98	ENSG00000136997,ENSG00000187098
15	GOTERM_BP_FAT	GO0042981	10.10	0.99	0.29	22.23	ENSG00000136997,ENSG00000187098,ENSG00000131323
16	GOTERM_BP_FAT	GO0043067	10.00	0.99	0.27	22.61	ENSG00000136997,ENSG00000187098,ENSG00000131323
17	GOTERM_BP_FAT	GO0010941	9.96	0.99	0.26	22.75	ENSG00000136997,ENSG00000187098,ENSG00000131323
18	KEGG_PATHWAY	hsa05200	9.30	0.36	0.36	15.88	ENSG00000136997,ENSG00000187098,ENSG00000131323
19	SP_PIR_KEYWORDS	nucleus	3.21	0.64	0.40	20.63	ENSG00000215301,ENSG00000136997,ENSG00000187098,ENSG00000129084,ENSG00000128513
20	GOTERM_MF_FAT	GO0043565	9.17	0.73	0.73	24.09	ENSG00000136997,ENSG00000187098,ENSG00000128513
21	GOTERM_BP_FAT	GO0051052	47.47	1.00	0.37	35.05	ENSG00000136997,ENSG00000128513

22	GOTERM_BP_FAT	GO0032844	47.47	1.00	0.37	35.05	ENSG00000136997,ENSG00000128513
23	UP_SEQ_FEATURE	domain	49.64	0.67	0.67	25.92	ENSG00000136997,ENSG00000187098
24	UP_SEQ_FEATURE	domain	47.90	0.68	0.44	26.72	ENSG00000136997,ENSG00000187098
25	GOTERM_CC_FAT	GO0031981	6.61	0.65	0.41	26.41	ENSG00000215301,ENSG00000136997,ENSG00000128513
26	GOTERM_CC_FAT	GO0016604	38.04	0.68	0.32	28.48	ENSG00000215301,ENSG00000136997
27	INTERPRO	IPR001092	41.75	0.63	0.63	28.15	ENSG00000136997,ENSG00000187098
28	SMART	SM00353	31.86	0.26	0.26	21.49	ENSG00000136997,ENSG00000187098
29	UP_SEQ_FEATURE	DNA-binding region	33.92	0.80	0.42	35.57	ENSG00000136997,ENSG00000187098
30	GOTERM_BP_FAT	GO0051345	30.23	1.00	0.50	49.30	ENSG00000136997,ENSG00000128513
31	GOTERM_CC_FAT	GO0070013	5.39	0.79	0.32	36.69	ENSG00000215301,ENSG00000136997,ENSG00000128513
32	KEGG_PATHWAY	hsa04622	28.65	0.66	0.41	34.40	ENSG00000215301,ENSG00000131323
33	GOTERM_CC_FAT	GO0043233	5.27	0.81	0.28	37.99	ENSG00000215301,ENSG00000136997,ENSG00000128513
34	GOTERM_CC_FAT	GO0031974	5.17	0.82	0.25	39.14	ENSG00000215301,ENSG00000136997,ENSG00000128513
35	GOTERM_BP_FAT	GO0033043	24.94	1.00	0.55	56.16	ENSG00000136997,ENSG00000128513
36	KEGG_PATHWAY	hsa05222	24.21	0.72	0.34	39.32	ENSG00000136997,ENSG00000131323
37	GOTERM_MF_FAT	GO0043566	25.58	0.95	0.78	46.95	ENSG00000136997,ENSG00000128513
38	GOTERM_BP_FAT	GO0007423	23.63	1.00	0.55	58.13	ENSG00000136997,ENSG00000187098
39	GOTERM_MF_FAT	GO0003677	3.18	0.97	0.69	52.12	ENSG00000215301,ENSG00000136997,ENSG00000187098,ENSG00000128513
40	GOTERM_BP_FAT	GO0043086	19.54	1.00	0.60	65.18	ENSG00000129084,ENSG00000128513
41	GOTERM_BP_FAT	GO0006917	16.91	1.00	0.64	70.50	ENSG00000136997,ENSG00000131323
42	GOTERM_BP_FAT	GO0012502	16.86	1.00	0.62	70.61	ENSG00000136997,ENSG00000131323

43	GOTERM_BP_FAT	GO0044092	16.20	1.00	0.62	72.05	ENSG00000129084,ENSG00000128513
44	SP_PIR_KEYWORDS	isopeptide bond	17.23	0.98	0.74	60.07	ENSG00000187098,ENSG00000129084
45	GOTERM_BP_FAT	GO0051336	16.06	1.00	0.61	72.38	ENSG00000136997,ENSG00000128513

Golden retriever B-cell lymphoma, top 15 significantly mutated genes

	Category	Term	Description	% input genes implicated	p-value	List Total	Pop Hits	Pop Total
1	GOTERM_BP_FAT	GO0051095	regulation of helicase activity	14.3	5.68E-03	12	7	13528
2	GOTERM_CC_FAT	GO0005829	cytosol	28.6	9.56E-03	6	1330	12782
3	SP_PIR_KEYWORDS	nucleus		57.1	1.28E-02	14	4283	19235
4	GOTERM_BP_FAT	GO0043086	negative regulation of catalytic activity	21.4	2.03E-02	12	277	13528
5	GOTERM_BP_FAT	GO0008156	negative regulation of DNA replication	14.3	2.25E-02	12	28	13528
6	BIOCARTA	h_rnaPathway	Double Stranded RNA Induced Gene Expression	14.3	2.48E-02	5	9	1437
7	GOTERM_BP_FAT	GO0044092	negative regulation of molecular function	21.4	2.89E-02	12	334	13528
8	GOTERM_BP_FAT	GO0051336	regulation of hydrolase activity	21.4	2.93E-02	12	337	13528
9	GOTERM_BP_FAT	GO0051053	negative regulation of DNA metabolic process	14.3	3.13E-02	12	39	13528
10	BIOCARTA	h_cd40Pathway	CD40L Signaling Pathway	14.3	3.84E-02	5	14	1437
11	BIOCARTA	h_tall1Pathway	TACI and BCMA stimulation of B cell immune responses	14.3	3.84E-02	5	14	1437
12	GOTERM_CC_FAT	GO0005654	nucleoplasm	21.4	4.13E-02	6	882	12782
13	UP_SEQ_FEATURE	mutagenesis site		35.7	4.24E-02	14	2045	19113
14	GOTERM_MF_FAT	GO0003697	single-stranded DNA binding	14.3	4.56E-02	12	55	12983
15	BIOCARTA	h_tnfr2Pathway	TNFR2 Signaling Pathway	14.3	4.65E-02	5	17	1437
16	SP_PIR_KEYWORDS	activator		21.4	4.67E-02	14	520	19235

17	GOTERM_BP_FAT	GO0006275	regulation of DNA replication	14.3	4.93E-02	12	62	13528
18	GOTERM_BP_FAT	GO0051276	chromosome organization	21.4	5.70E-02	12	485	13528
19	GOTERM_CC_FAT	GO0016604	nuclear body	14.3	6.40E-02	6	168	12782
20	GOTERM_BP_FAT	GO0043085	positive regulation of catalytic activity	21.4	6.45E-02	12	520	13528
21	GOTERM_BP_FAT	GO0043632	modification-dependent macromolecule catabolic process	21.4	7.67E-02	12	574	13528
22	GOTERM_BP_FAT	GO0019941	modification-dependent protein catabolic process	21.4	7.67E-02	12	574	13528
23	GOTERM_BP_FAT	GO0044093	positive regulation of molecular function	21.4	7.95E-02	12	586	13528
24	GOTERM_BP_FAT	GO0051603	proteolysis involved in cellular protein catabolic process	21.4	8.29E-02	12	600	13528
25	GOTERM_BP_FAT	GO0044257	cellular protein catabolic process	21.4	8.36E-02	12	603	13528
26	GOTERM_BP_FAT	GO0007568	aging	14.3	8.59E-02	12	110	13528
27	GOTERM_BP_FAT	GO0030163	protein catabolic process	21.4	8.82E-02	12	622	13528
28	GOTERM_BP_FAT	GO0051052	regulation of DNA metabolic process	14.3	8.89E-02	12	114	13528
29	SP_PIR_KEYWORDS	acetylation		35.7	9.07E-02	14	2635	19235
30	BBID	99	NF-kB_activation	14.3	9.28E-02	3	17	358

Golden retriever B-cell lymphoma, continued

	Category	Term	Fold Enrichment	Bonferroni	Benjamini	FDR	Genes
1	GOTERM_BP_FAT	GO0051095	322.10	0.85	0.85	7.45	*ENSG00000141510,ENSG00000128513*
2	GOTERM_CC_FAT	GO0005829	6.41	0.34	0.34	8.56	*ENSG00000198242,ENSG00000141510,ENSG00000129084,ENSG00000006062*
3	SP_PIR_KEYWORDS	nucleus	2.57	0.55	0.55	12.20	*ENSG00000215301,ENSG00000109670,ENSG00000141510,ENSG00000129084,ENSG00000100*
4	GOTERM_BP_FAT	GO0043086	12.21	1.00	0.97	24.36	*ENSG00000141510,ENSG00000129084,ENSG00000128513*
5	GOTERM_BP_FAT	GO0008156	80.52	1.00	0.92	26.64	*ENSG00000141510,ENSG00000128513*
6	BIOCARTA	h_rnaPathway	63.87	0.55	0.55	19.58	*ENSG00000141510,ENSG00000006062*

7	GOTERM_BP_FAT	GO0044092	10.13	1.00	0.91	32.82	*ENSG00000141510,ENSG00000129084,ENSG00000128513*
8	GOTERM_BP_FAT	GO0051336	10.04	1.00	0.86	33.27	*ENSG00000214946,ENSG00000141510,ENSG00000128513*
9	GOTERM_BP_FAT	GO0051053	57.81	1.00	0.83	35.06	*ENSG00000141510,ENSG00000128513*
10	BIOCARTA	h_cd40Pathway	41.06	0.71	0.47	28.79	*ENSG00000131323,ENSG00000006062*
11	BIOCARTA	h_tall1Pathway	41.06	0.71	0.47	28.79	*ENSG00000131323,ENSG00000006062*
12	GOTERM_CC_FAT	GO0005654	7.25	0.84	0.60	32.49	*ENSG00000215301,ENSG00000141510,ENSG00000128513*
13	UP_SEQ_FEATURE	mutagenesis site	3.34	0.96	0.96	36.46	*ENSG00000215301,ENSG00000141510,ENSG00000181555,ENSG00000146278,ENSG00000006*
14	GOTERM_MF_FAT	GO0003697	39.34	0.98	0.98	39.29	*ENSG00000141510,ENSG00000128513*
15	BIOCARTA	h_tnfr2Pathway	33.81	0.78	0.40	33.81	*ENSG00000131323,ENSG00000006062*
16	SP_PIR_KEYWORDS	activator	7.93	0.95	0.77	38.20	*ENSG00000141510,ENSG00000181555,ENSG00000146278*
17	GOTERM_BP_FAT	GO0006275	36.37	1.00	0.91	49.68	*ENSG00000141510,ENSG00000128513*
18	GOTERM_BP_FAT	GO0051276	6.97	1.00	0.91	54.92	*ENSG00000141510,ENSG00000181555,ENSG00000128513*
19	GOTERM_CC_FAT	GO0016604	25.36	0.94	0.61	45.97	*ENSG00000215301,ENSG00000141510*
20	GOTERM_BP_FAT	GO0043085	6.50	1.00	0.92	59.57	*ENSG00000141510,ENSG00000129084,ENSG00000128513*
21	GOTERM_BP_FAT	GO0043632	5.89	1.00	0.93	66.19	*ENSG00000120833,ENSG00000109670,ENSG00000129084*
22	GOTERM_BP_FAT	GO0019941	5.89	1.00	0.93	66.19	*ENSG00000120833,ENSG00000109670,ENSG00000129084*
23	GOTERM_BP_FAT	GO0044093	5.77	1.00	0.92	67.57	*ENSG00000141510,ENSG00000129084,ENSG00000128513*

24	GOTERM_BP_FAT	GO0051603	5.64	1.00	0.91	69.13	*ENSG00000120833,ENSG00000109670,ENSG00000129084*
25	GOTERM_BP_FAT	GO0044257	5.61	1.00	0.89	69.46	*ENSG00000120833,ENSG00000109670,ENSG00000129084*
26	GOTERM_BP_FAT	GO0007568	20.50	1.00	0.88	70.50	*ENSG00000120833,ENSG00000141510*
27	GOTERM_BP_FAT	GO0030163	5.44	1.00	0.87	71.48	*ENSG00000120833,ENSG00000109670,ENSG00000129084*
28	GOTERM_BP_FAT	GO0051052	19.78	1.00	0.86	71.79	*ENSG00000141510,ENSG00000128513*
29	SP_PIR_KEYWORDS	acetylation	2.61	1.00	0.86	61.55	*ENSG00000215301,ENSG00000198242,ENSG00000141510,ENSG00000129084,ENSG00000100*
30	BBID	99	14.04	0.54	0.54	41.34	*ENSG00000141510,ENSG00000006062*

Boxer T-cell lymphoma

	Category	Term	Description	% input genes implicated	p-value	List Total	Pop Hits	Pop Total
1	UP_SEQ_FEATURE	mutagenesis site		80.0	4.50E-03	5	2045	19113
2	KEGG_PATHWAY	hsa05213	Endometrial cancer	40.0	1.02E-02	2	52	5085
3	KEGG_PATHWAY	hsa05214	Glioma	40.0	1.24E-02	2	63	5085
4	GOTERM_BP_FAT	GO0007162	negative regulation of cell adhesion	40.0	1.27E-02	5	43	13528
5	KEGG_PATHWAY	hsa05218	Melanoma	40.0	1.40E-02	2	71	5085
6	GOTERM_BP_FAT	GO0051291	protein heterooligomerization	40.0	1.53E-02	5	52	13528
7	KEGG_PATHWAY	hsa05215	Prostate cancer	40.0	1.75E-02	2	89	5085
8	BIOCARTA	h_metPathway	Signaling of Hepatocyte Growth Factor Receptor	40.0	2.37E-02	2	34	1437
9	GOTERM_CC_FAT	GO0030425	dendrite	40.0	2.53E-02	3	163	12782

10	GOTERM_CC_FAT	GO0043025	cell soma	40.0	2.61E-02	3	168	12782
11	GOTERM_MF_FAT	GO0004725	protein tyrosine phosphatase activity	40.0	3.17E-02	5	104	12983
12	GOTERM_CC_FAT	GO0044463	cell projection part	40.0	3.63E-02	3	234	12782
13	KEGG_PATHWAY	hsa04510	Focal adhesion	40.0	3.95E-02	2	201	5085
14	GOTERM_BP_FAT	GO0030155	regulation of cell adhesion	40.0	3.99E-02	5	137	13528
15	GOTERM_BP_FAT	GO0030334	regulation of cell migration	40.0	4.90E-02	5	169	13528
16	GOTERM_MF_FAT	GO0004721	phosphoprotein phosphatase activity	40.0	4.99E-02	5	165	12983
17	GOTERM_BP_FAT	GO0051259	protein oligomerization	40.0	5.05E-02	5	174	13528
18	GOTERM_CC_FAT	GO0043005	neuron projection	40.0	5.28E-02	3	342	12782
19	GOTERM_BP_FAT	GO0040012	regulation of locomotion	40.0	5.56E-02	5	192	13528
20	GOTERM_BP_FAT	GO0048545	response to steroid hormone stimulus	40.0	5.56E-02	5	192	13528
21	GOTERM_BP_FAT	GO0051270	regulation of cell motion	40.0	5.59E-02	5	193	13528
22	KEGG_PATHWAY	hsa05200	Pathways in cancer	40.0	6.45E-02	2	328	5085
23	GOTERM_BP_FAT	GO0031175	neuron projection development	40.0	7.36E-02	5	256	13528
24	GOTERM_MF_FAT	GO0016791	phosphatase activity	40.0	7.45E-02	5	249	12983
25	SP_PIR_KEYWORDS	acetylation		60.0	9.31E-02	5	2635	19235
26	GOTERM_BP_FAT	GO0048666	neuron development	40.0	9.65E-02	5	339	13528
27	GOTERM_BP_FAT	GO0045859	regulation of protein kinase activity	40.0	9.82E-02	5	345	13528

Boxer T-cell lymphoma, continued

	Category	Term	Fold Enrichment	Bonferroni	Benjamini	FDR	Genes
1	UP_SEQ_FEATURE	mutagenesis site	7.48	0.20	0.20	4.25	*ENSG00000184408,ENSG00000171862,ENSG00000169032,ENSG00000182568*
2	KEGG_PATHWAY	hsa05213	97.79	0.35	0.35	9.07	*ENSG00000171862,ENSG00000169032*
3	KEGG_PATHWAY	hsa05214	80.71	0.41	0.23	10.90	*ENSG00000171862,ENSG00000169032*
4	GOTERM_BP_FAT	GO0007162	125.84	0.96	0.96	15.34	*ENSG00000171862,ENSG00000169032*

5	KEGG_PATHWAY	hsa05218	71.62	0.45	0.18	12.20	*ENSG00000171862,ENSG00000169032*
6	GOTERM_BP_FAT	GO0051291	104.06	0.98	0.87	18.25	*ENSG00000184408,ENSG00000169032*
7	KEGG_PATHWAY	hsa05215	57.13	0.52	0.17	15.08	*ENSG00000171862,ENSG00000169032*
8	BIOCARTA	h_metPathway	42.26	0.70	0.70	20.60	*ENSG00000171862,ENSG00000169032*
9	GOTERM_CC_FAT	GO0030425	52.28	0.65	0.65	21.04	*ENSG00000184408,ENSG00000169032*
10	GOTERM_CC_FAT	GO0043025	50.72	0.66	0.42	21.61	*ENSG00000184408,ENSG00000169032*
11	GOTERM_MF_FAT	GO0004725	49.93	0.89	0.89	28.18	*ENSG00000171862,ENSG00000169032*
12	GOTERM_CC_FAT	GO0044463	36.42	0.78	0.40	28.83	*ENSG00000184408,ENSG00000169032*
13	KEGG_PATHWAY	hsa04510	25.30	0.82	0.29	31.15	*ENSG00000171862,ENSG00000169032*
14	GOTERM_BP_FAT	GO0030155	39.50	1.00	0.97	41.29	*ENSG00000171862,ENSG00000169032*
15	GOTERM_BP_FAT	GO0030334	32.02	1.00	0.96	48.20	*ENSG00000171862,ENSG00000169032*
16	GOTERM_MF_FAT	GO0004721	31.47	0.97	0.82	40.93	*ENSG00000171862,ENSG00000169032*
17	GOTERM_BP_FAT	GO0051259	31.10	1.00	0.93	49.20	*ENSG00000184408,ENSG00000169032*
18	GOTERM_CC_FAT	GO0043005	24.92	0.89	0.43	39.30	*ENSG00000184408,ENSG00000169032*
19	GOTERM_BP_FAT	GO0040012	28.18	1.00	0.92	52.66	*ENSG00000171862,ENSG00000169032*
20	GOTERM_BP_FAT	GO0048545	28.18	1.00	0.92	52.66	*ENSG00000171862,ENSG00000169032*
21	GOTERM_BP_FAT	GO0051270	28.04	1.00	0.88	52.85	*ENSG00000171862,ENSG00000169032*
22	KEGG_PATHWAY	hsa05200	15.50	0.94	0.37	46.05	*ENSG00000171862,ENSG00000169032*
23	GOTERM_BP_FAT	GO0031175	21.14	1.00	0.92	63.20	*ENSG00000171862,ENSG00000169032*
24	GOTERM_MF_FAT	GO0016791	20.86	0.99	0.83	54.93	*ENSG00000171862,ENSG00000169032*
25	SP_PIR_KEYWORDS	acetylation	4.38	0.99	0.99	61.29	*ENSG00000171862,ENSG00000169032,ENSG00000182568*
26	GOTERM_BP_FAT	GO0048666	15.96	1.00	0.95	73.50	*ENSG00000171862,ENSG00000169032*
27	GOTERM_BP_FAT	GO0045859	15.68	1.00	0.93	74.12	*ENSG00000171862,ENSG00000169032*

Golden retriever T-cell lymphoma, top 15 significantly mutated genes

	Category	Term	Description	% input genes implicated	p-value	List Total	Pop Hits	Pop Total
1	SP_PIR_KEYWORDS	acetylation		53.3	1.27E-03	15	2635	19235
2	GOTERM_CC_FAT	GO0005829	cytosol	33.3	9.57E-03	10	1330	12782
3	SP_PIR_KEYWORDS	rrna-binding		13.3	1.23E-02	15	17	19235
4	SP_PIR_KEYWORDS	mitochondrion		26.7	2.05E-02	15	832	19235
5	GOTERM_CC_FAT	GO0022625	cytosolic large ribosomal subunit	13.3	2.64E-02	10	38	12782
6	GOTERM_MF_FAT	GO0019843	rRNA binding	13.3	2.65E-02	13	29	12983
7	GOTERM_CC_FAT	GO0005739	mitochondrion	26.7	3.49E-02	10	1087	12782
8	GOTERM_CC_FAT	GO0015934	large ribosomal subunit	13.3	4.62E-02	10	67	12782
9	GOTERM_CC_FAT	GO0030529	ribonucleoprotein complex	20.0	4.83E-02	10	515	12782
10	GOTERM_CC_FAT	GO0022626	cytosolic ribosome	13.3	5.56E-02	10	81	12782
11	GOTERM_CC_FAT	GO0044429	mitochondrial part	20.0	6.27E-02	10	595	12782
12	GOTERM_BP_FAT	GO0009611	response to wounding	20.0	6.67E-02	12	530	13528
13	GOTERM_BP_FAT	GO0006414	translational elongation	13.3	7.92E-02	12	101	13528
14	GOTERM_MF_FAT	GO0015078	hydrogen ion	13.3	8.01E-02	13	90	12983
15	GOTERM_CC_FAT	GO0033279	ribosomal subunit	13.3	8.66E-02	10	128	12782
16	GOTERM_MF_FAT	GO0015077	monovalent inorganic cation	13.3	9.20E-02	13	104	12983

Golden retriever T-cell lymphoma, top 15 significantly mutated genes, continued

	Category	Term	Fold Enrichment	Bonferroni	Benjamini	FDR	Genes
1	SP_PIR_KEYWORDS	acetylation	3.89	0.09	0.09	1.32	*ENSG00000111679,ENSG00000182890,ENSG00000142676,ENSG00000198242,ENSG00000111*
2	GOTERM_CC_FAT	GO0005829	4.81	0.39	0.39	8.88	*ENSG00000111679,ENSG00000142676,ENSG00000198242,ENSG00000129084,ENSG00000171*

3	SP_PIR_KEYWORDS	rrna-binding	150.86	0.59	0.36	12.09	*ENSG00000142676,ENSG00000198242*
4	SP_PIR_KEYWORDS	mitochondrion	6.17	0.78	0.39	19.42	*ENSG00000176340,ENSG00000182890,ENSG00000171487,ENSG00000167863*
5	GOTERM_CC_FAT	GO0022625	67.27	0.75	0.50	22.84	*ENSG00000142676,ENSG00000198242*
6	GOTERM_MF_FAT	GO0019843	68.88	0.85	0.85	24.26	*ENSG00000142676,ENSG00000198242*
7	GOTERM_CC_FAT	GO0005739	4.70	0.84	0.45	29.08	*ENSG00000176340,ENSG00000182890,ENSG00000171487,ENSG00000167863*
8	GOTERM_CC_FAT	GO0015934	38.16	0.91	0.45	36.73	*ENSG00000142676,ENSG00000198242*
9	GOTERM_CC_FAT	GO0030529	7.45	0.92	0.40	38.08	*ENSG00000142676,ENSG00000198242,ENSG00000129084*
10	GOTERM_CC_FAT	GO0022626	31.56	0.95	0.39	42.52	*ENSG00000142676,ENSG00000198242*
11	GOTERM_CC_FAT	GO0044429	6.44	0.96	0.38	46.53	*ENSG00000176340,ENSG00000182890,ENSG00000167863*
12	GOTERM_BP_FAT	GO0009611	6.38	1.00	1.00	58.01	*ENSG00000111679,ENSG00000111144,ENSG00000169403*
13	GOTERM_BP_FAT	GO0006414	22.32	1.00	1.00	64.55	*ENSG00000142676,ENSG00000198242*
14	GOTERM_MF_FAT	GO0015078	22.19	1.00	0.95	57.86	*ENSG00000176340,ENSG00000167863*
15	GOTERM_CC_FAT	GO0033279	19.97	0.99	0.44	58.38	*ENSG00000142676,ENSG00000198242*
16	GOTERM_MF_FAT	GO0015077	19.21	1.00	0.89	63.18	*ENSG00000176340,ENSG00000167863*
