# Supplementary material for: Exome sequencing of lymphomas from three dog breeds reveals somatic mutation patterns reflecting genetic background
Source: Genome Res. 2015 Nov;25(11):1634–45. doi: 10.1101/gr.194449.115 (PMC4617960; doi:10.1101/gr.194449.115)
Supplement: Supplemental Material [file supp_gr.194449.115_Supp_Table9.pdf]

Supplementary Table 9. Networks suggested by IPA for significantly mutated genes from B-cell lymphomas from boxer and golden retriever

Boxer

Network ID	Molecules in Network	Score	Focus Molecules	Top Diseases and Functions
1	1,3,4,5-IP4,ABTB1,ARHGAP5,BDKRB1,CA3,CCDC6,Ck2, dihydrosphingosine 1-phosphate,EEF1A1,EMILIN1,ERK1/2, FGF23,GFRA2,H2BFM,HADH,HCLS1,Insulin,KCND2,MAP2K1, MAST2,mir-25,mir-26,mir-148,miR-199a-3p (and other miRNAs w/seed CAGUAGU),miR-26a-5p (and other miRNAs w/seed UCAAGUA),MTORC2,NEU1,PKC alpha/beta,Pkc(s), PSCA,PTEN,PTPRZ1,SATB1,TNFAIP1,VEGFB	14	5	Cellular Development, Cellular Growth and Proliferation, Cellular Movement
2	NALP,NLR,NLRP14	3	1	Cellular Development, Cellular Growth and Proliferation, Embryonic Development

Golden retriever

Network ID	Molecules in Network	Score	Focus Molecules	Top Diseases and Functions
1	ABCD3,ACTR6,AGRP,ATP5H,beta-estradiol,C11orf49,COX8A, FBXW7,GLUD1,HNRNPA1L2,KRTAP10-6,LDOC1,LTA4H, MAPRE3,MC4R,MRFAP1L1,MT-CO2,NLRP5,NME7,PAAF1, POMP,PSMA1,PSMA8,PSMB2,PTAFR,PTPN6,RAD54B,RPL23A, SATB1,SH3BP4,TXNL1,UBC,ZFAND2A,ZFAND2B,ZNF706	44	15	Connective Tissue Development and Function, Tissue Morphology, Cell-To-Cell Signaling and Interaction
2	ANG,Arf,AURKA,C12orf5,COPS6,CUL1,CUL4B,EEF2,IGF1R,IGF 2BP1,IPO7,MAGEC2,miR-542-3p (miRNAs w/seed GUGACAG),MYBBP1A,MYC,NBR1,NEDD8,NUB1,PDCD4,POLR 1A,PSMD4,RAD23B,RPL5,RPL11,RPL23,RPL26,RPL7A,RPS3, SMG1,SNCA,TP53,UBC,UBIQUITIN LIGASE,UBQLN1	4	2	Protein Synthesis, Cell Cycle, Developmental Disorder
