# Supplementary material for: Exome sequencing of lymphomas from three dog breeds reveals somatic mutation patterns reflecting genetic background
Source: Genome Res. 2015 Nov;25(11):1634–45. doi: 10.1101/gr.194449.115 (PMC4617960; doi:10.1101/gr.194449.115)
Supplement: Supplemental Material [file supp_gr.194449.115_Supplemental_Figures.docx]

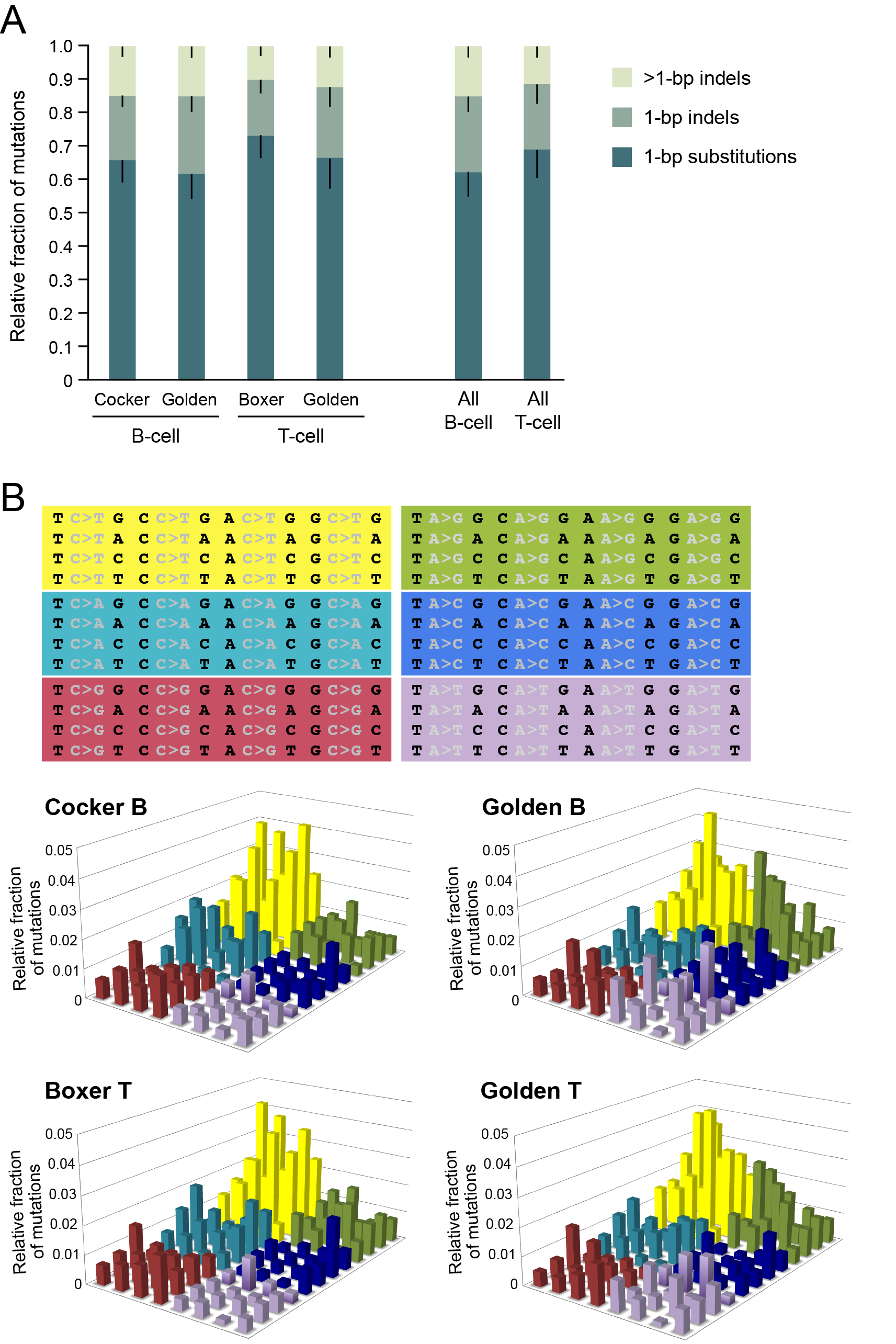


**Supplementary Figure 1. General mutation load in B- and T-cell lymphoma.** **A,** relative fraction of mutation type show overall similarities but with relatively more larger indels and less single basepair substitutions in B-cell lymphomas. **B,** single base-pair substitutions plotted to visualize the preceding and following base. The single base-pair substitution pattern is similar in all sample groups, with certain substitution patterns over- or underrepresented.


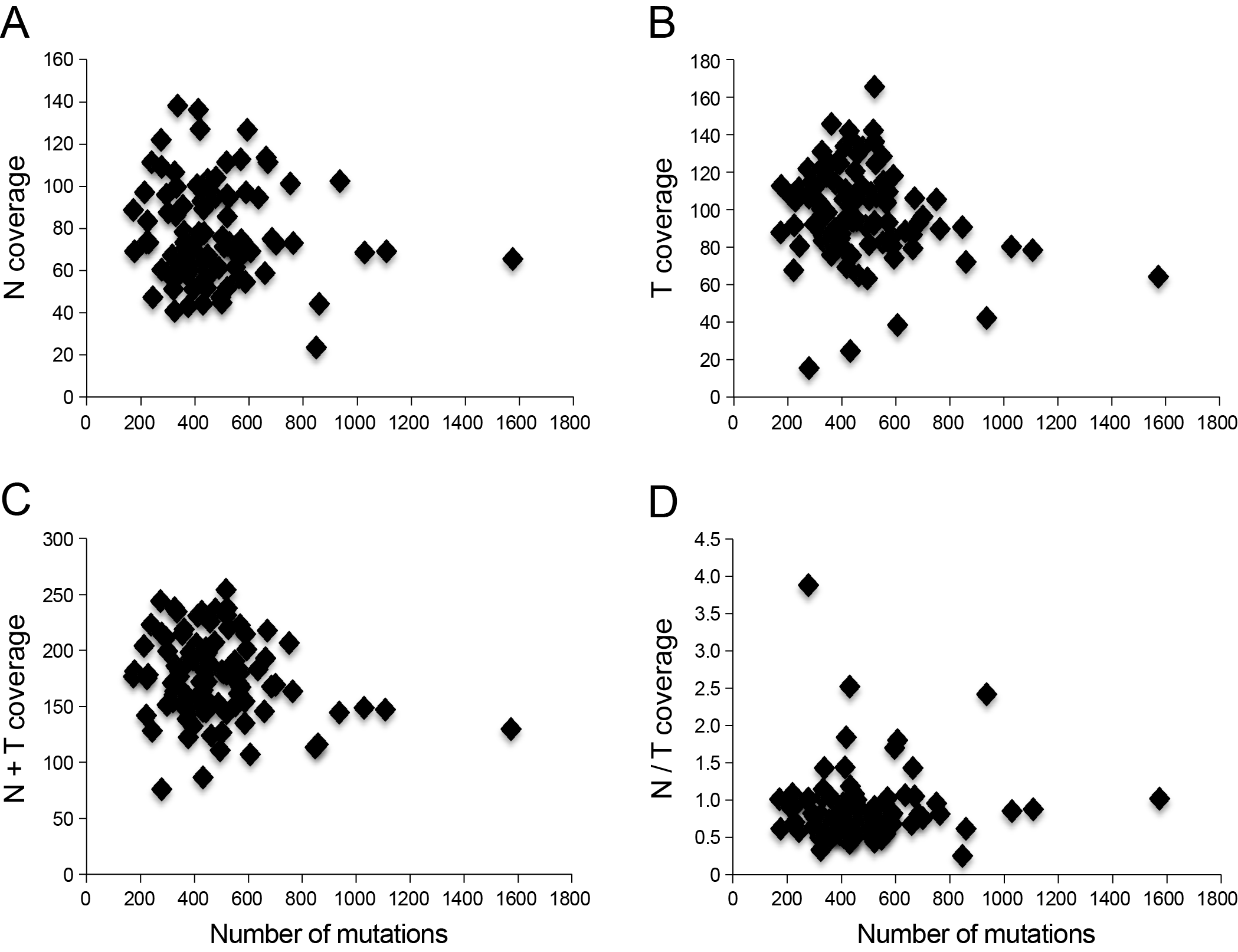


**Supplementary Figure 2. Sequencing depth (y-axis) does not correlate with number of tumor-specific mutations (x-axis)** when comparing mutations to **A.** Normal coverage, **B.** Tumor coverage, **C.** Tumor+Normal coverage, or **D.** Normal/Tumor coverage.

**
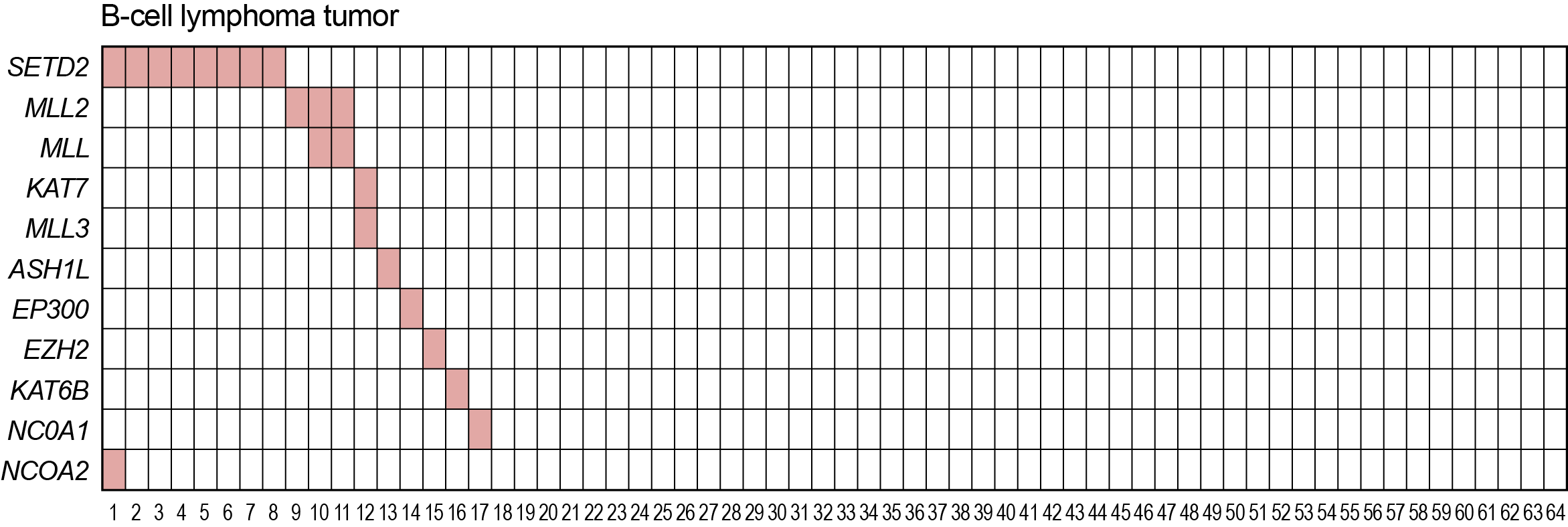
**

**Supplementary Figure 3. Histone acetyltransferase and methyltransferase mutations** in B-cell lymphoma tumors. 12.5% of all B-cell lymphomas studied have a mutation in *SETD1*. Red, mutation. White, no mutation.

**
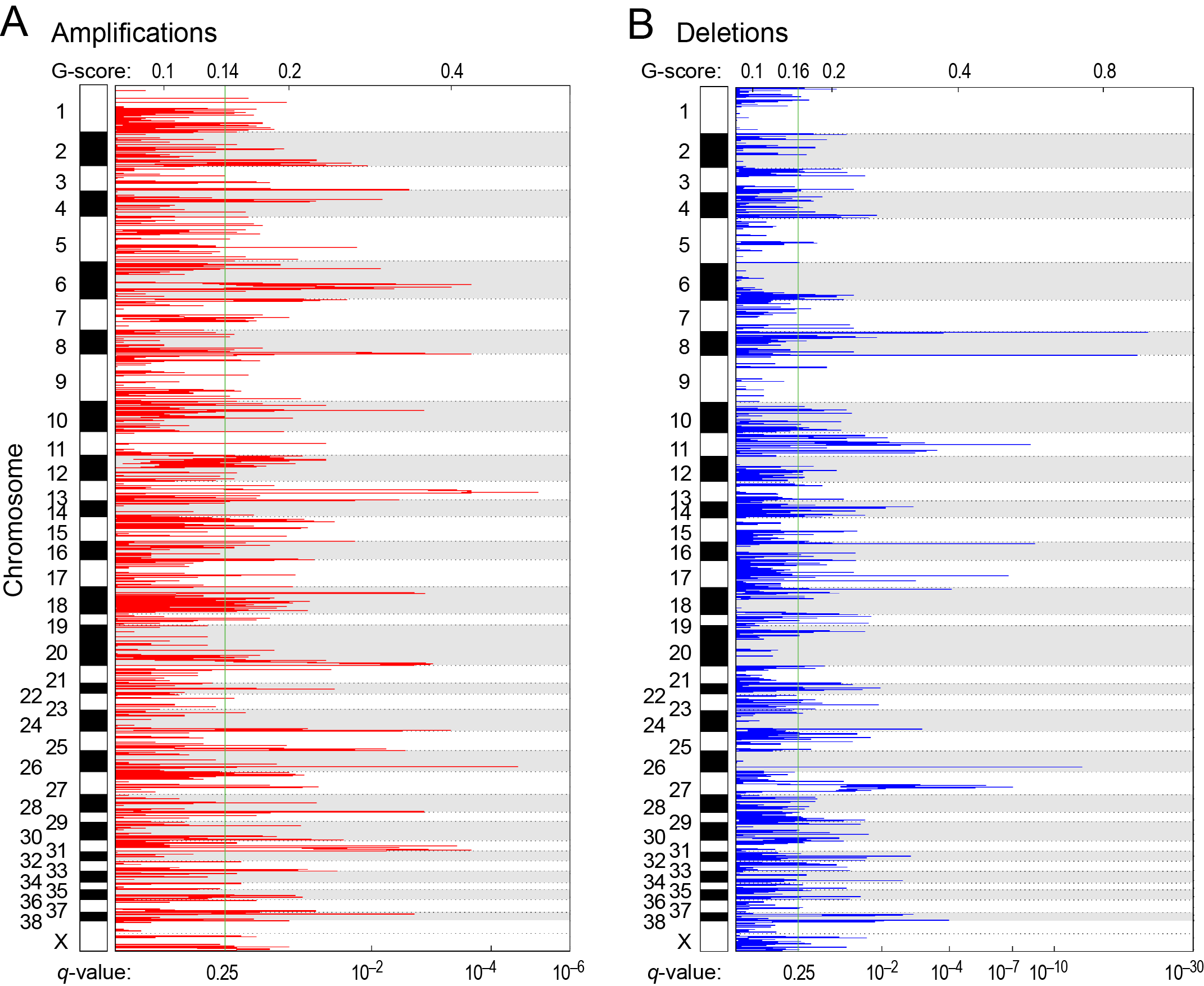
**

**Supplementary Figure 4. Somatic copy number alterations** estimated with GISTIC visualizing **A.** amplifications, and **B.** deletions.
