# Supplementary material for: Exome sequencing of lymphomas from three dog breeds reveals somatic mutation patterns reflecting genetic background
Source: Genome Res. 2015 Nov;25(11):1634–45. doi: 10.1101/gr.194449.115 (PMC4617960; doi:10.1101/gr.194449.115)
Supplement: Supplemental Material [file supp_gr.194449.115_Supplemental_Information.docx]

**Supplementary Information. Pathology information for analyzed samples.**

**Implicated lymph nodes.** Generally, canine lymphomas have multicentric presentation. The clinical progression of disease in stage II (limited to lymph nodes on one side of the diaphragm), stage III (lymph nodes affected on both sides of the diagram), and stage IV (extranodal disease) is comparable. The morphological features and IHC characteristics of the disease is indistinguishable when multiple lymph nodes are sampled in the same dog. Diagnostic samples are usually obtained from the most accessible lymph nodes, including the submandibular, or the prescapular, or the popliteal chains. Previous data using replicate samples from multiple lymph nodes showed that the anatomical location did not drive differences in gene expression for lymphoma, and there was no variation in gene expression from samples obtained from different lymph nodes in the same dog (Frantz et al, Vet Pathol. 2013 50(4): 693-703). The anatomical location of lymph nodes from which the samples in this study originated is therefore not predicted to influence the mutational status of the tumor.

Pathology reports were available for 67% of all cases, and in 66% (n=46) of the reports, the lymph node was specified. Among those, 63% were popliteal, 22% were prescapular, and 15% were either submandibular or multiple lymph nodes. No significant difference in implicated lymph node was seen between breeds.

**Familial history of lymphomas.** While there are clear breed predispositions for lymphoma, and even for different types of lymphoma, there is no evidence to date that indicates the disease within breeds is distributed along familial lines. The observations of long latency of disease (median age of onset is approximately 8 years, which is late adulthood), occurrence in related siblings or parent/sibling pairs does not seem to be more frequent than in breeds as a whole, and dominant germline mutations that inevitably lead to disease phenotype have not been defined, indicate that canine lymphoma is a sporadic disease albeit with genetic predisposing factors.

**Sample subtype details.** Immunophenotyping information was either supplied with the sample or determined, as described in Methods. Subtyping information was available for one third of the samples:

**Supplementary Table 11.** Subtyping information where available

|  | fraction known | diagnosis of those known |
| --- | --- | --- |
| cocker B | 1 of 10 | 1 DLBCL |
| golden B | 21 of 54 | 15 DLBCL, 5 MZL, 1 BKL |
| boxer T | 6 of 16 | 4 PTCL, 1 CTCL, 1 LBT |
| golden T | 7 of 25 | 4 TZL, 2 PTCL, 1 CTCL |

Cases from the same breed with different subtypes were scanned for shared mutated genes. Examples of genes (canine Ensembl IDs) are given below, where ‘x’ indicates a mutation in said gene. Due to the number of different subtypes present in each breed and the number of individuals with missing subtypes, it is impossible to say if the genetic differences between subtypes in the same breed are smaller or larger than the genetic difference between tumors from different breeds.

**Supplementary Table 12.** Examples of individuals from the same breed, with different subtypes, sharing mutated genes.

| patient | breed | B/T | subtype | ENSCAFG00000019735 |  |  |  |
| --- | --- | --- | --- | --- | --- | --- | --- |
| case 1 | boxer | T | LBT | x |  |  |  |
| case 2 | boxer | T | PTCL | x |  |  |  |
|  |  |  |  |  |  |  |  |
| patient | breed | B/T | subtype | ENSCAFG00000031638 | ENSCAFG00000002501 | ENSCAFG00000000584 |  |
| case 3 | golden | T | PTCL | x | x | x |  |
| case 4 | golden | T | TZL | x | x | x |  |
|  |  |  |  |  |  |  |  |
| patient | breed | B/T | subtype | ENSCAFG00000031638 | ENSCAFG00000017753 | ENSCAFG00000030674 | ENSCAFG00000006732 |
| case 5 | golden | B | DLBCL |  | x |  |  |
| case 6 | golden | B | DLBCL | x |  |  | x |
| case 7 | golden | B | DLBCL |  | x |  |  |
| case 8 | golden | B | DLBCL | x |  |  |  |
| case 9 | golden | B | DLBCL |  |  | x |  |
| case 10 | golden | B | MZL | x | x |  |  |
| case 11 | golden | B | MZL |  |  | x | x |
